# Contrasting responses of non-small cell lung cancer to antiangiogenic therapies depend on histological subtype

**DOI:** 10.1002/emmm.201303214

**Published:** 2014-02-05

**Authors:** Marta Larrayoz, Ruben Pio, María J Pajares, Isabel Zudaire, Daniel Ajona, Oriol Casanovas, Luis M Montuenga, Jackeline Agorreta

**Affiliations:** 1 Division of Oncology, Center for Applied Medical ResearchPamplona, Spain; 2 Department of Histology and Pathology, School of Medicine, University of NavarraPamplona, Spain; 3 Department of Biochemistry and Genetics, School of Sciences, University of NavarraPamplona, Spain; 4 Translational Research Laboratory, Catalan Institute of Oncology, IDIBELL, L'Hospitalet de LlobregatBarcelona, Spain

**Keywords:** angiogenesis, lung cancer, mouse models, *N*-nitroso-tris-chloroethylurea, squamous cell carcinoma

## Abstract

The vascular endothelial growth factor (VEGF) pathway is a clinically validated antiangiogenic target for non-small cell lung cancer (NSCLC). However, some contradictory results have been reported on the biological effects of antiangiogenic drugs. In order to evaluate the efficacy of these drugs in NSCLC histological subtypes, we analyzed the anticancer effect of two anti-VEGFR2 therapies (sunitinib and DC101) in chemically induced mouse models and tumorgrafts of lung adenocarcinoma (ADC) and squamous cell carcinoma (SCC). Antiangiogenic treatments induced vascular trimming in both histological subtypes. In ADC tumors, vascular trimming was accompanied by tumor stabilization. In contrast, in SCC tumors, antiangiogenic therapy was associated with disease progression and induction of tumor proliferation. Moreover, in SCC, anti-VEGFR2 therapies increased the expression of stem cell markers such as aldehyde dehydrogenase 1A1, CD133, and CD15, independently of intratumoral hypoxia. *In vitro* studies with ADC cell lines revealed that antiangiogenic treatments reduced pAKT and pERK signaling and inhibited proliferation, while in SCC-derived cell lines the same treatments increased pAKT and pERK, and induced survival. In conclusion, this study evaluates for the first time the effect of antiangiogenic drugs in lung SCC murine models *in vivo* and sheds light on the contradictory results of antiangiogenic therapies in NSCLC.

## Introduction

Lung cancer is the third leading cause of death in high-income countries (Lopez *et al*, 2006), and it remains the leading cause of cancer-related death worldwide (Siegel *et al*, 2013) with about 1.4 million deaths annually across the world (Jemal *et al*, 2011). Non-small cell lung cancer (NSCLC) is the most common type of lung cancer accounting for approximately 85% of lung cancers. NSCLC can be subdivided into two main histological subtypes: adenocarcinoma (ADC) and squamous cell carcinoma (SCC) that account for 50 and 30% of NSCLC cases, respectively (Langer *et al*, 2010). In the clinical practice, advanced NSCLC can be treated with cytotoxic chemotherapy in combination with targeted agents that block two main molecular pathways: cell signaling mediated by the epithelial growth factor receptor (EGFR) and angiogenesis mediated by the vascular endothelial growth factor (VEGF). The efficacy and safety of antiangiogenic therapies in NSCLC appear to be closely associated with the histological subtype of the tumor. Hence, the administration of bevacizumab, an anti-VEGF monoclonal antibody, is restricted to non-squamous patients (Johnson *et al*, 2004). However, the clinical benefit of bevacizumab is modest and episodes of resistance have been reported (Ebos & Kerbel, 2011). Furthermore, preclinical studies on pancreatic carcinoma, breast cancer, melanoma, and glioblastoma have evidenced a detrimental role of antiangiogenic agents related to an increase in their metastatic potential (Ebos *et al*, 2009; Paez-Ribes *et al*, 2009; Sennino *et al*, 2012). In an effort to develop more effective treatments, new antiangiogenic agents, such as the multi-tyrosine kinase inhibitors sunitinib and sorafenib, are under clinical evaluation (Reckamp, 2012). Interestingly, the phase III ESCAPE trial demonstrated greater mortality rates in SCC patients of the sorafenib arm (Scagliotti *et al*, 2010). Moreover, we recently reported data on the prognostic role of the VEGF pathway in early ADC and SCC of the lung, which stress the different behavior of both histologies. Specifically, we showed that high combined expression of VEGF, VEGFR1, and VEGFR2 in tumor cells is associated with good prognosis in early-stage SCC patients, but not in ADC (Pajares *et al*, 2012). Currently, there are hundreds of clinical trials under way to assess the use of VEGF-pathway-targeted drugs in a variety of tumors, and thousands of lung cancer patients are expected to be enrolled in these clinical trials in the next 5 years (http://clinicaltrials.gov). In these circumstances, a rigorous assessment of the significance of the VEGF pathway on the two main histologies of NSCLC and the biological response to antiangiogenic drugs in NSCLC is mandatory. For this purpose, mouse models of NSCLC are especially useful since the efficacy of new drugs can be tested in lung cancer tumors of specific histology. In addition, mouse models can be used simultaneously to human clinical trials following the rationale of co-clinical trials (Chen *et al*, 2012).

In the present study, we evaluated the efficacy of two anti-VEGFR2 compounds (sunitinib and DC101) on chemically induced mouse models of specific lung cancer subtypes: the urethane-induced mouse model of pulmonary ADC and the *N*-nitroso-tris-chloroethylurea (NTCU)-induced mouse model of lung SCC. We also analyzed the impact of VEGFR2 blockade on ADC and SCC tumorgrafts. Analyses of angiogenesis, proliferation, apoptosis, and stem cell marker expression demonstrated contrasting effects of antiangiogenic drugs in these two histological subtypes.

## Results

### Chemically induced mouse tumor models recapitulate specific histological subtypes of human lung cancer

Molecular and histopathological analysis of the urethane-induced model of pulmonary ADC and the NTCU-induced model of SCC was performed. The ADC model elicits neoplastic lesions with glandular differentiation and immunohistochemical staining of TTF-1 that recapitulate the histopathological features of human lung ADC (Supplementary Figs S1A,C,E). In the NTCU model, lung lesions were centrally located and frequently associated with bronchioles. Similarly to human SCC, intercellular bridges and keratin pearls could be observed (Supplementary Fig S1B). The squamous differentiation was confirmed by p63 staining (Supplementary Figs S1D,F). Both ADC and SCC tumors expressed VEGF and VEGFR2 (Supplementary Fig S2A).

### Differential tumor response to VEGFR2 inhibition in ADC and SCC

To determine the antitumor effect of anti-VEGFR therapies, mice bearing urethane-induced ADC or NTCU-induced SCC tumors were treated with sunitinib, a multi-tyrosine kinase inhibitor that blocks VEGF receptors. As sunitinib action is not restricted to VEGF receptors, we also tested the effect of a specific blocking antibody against murine VEGFR2 (DC101). The blockade of VEGFR2 by these antiangiogenic drugs was verified by the decrease in pVEGFR2 staining in endothelial and tumor cells (Supplementary Fig S2B). Furthermore, the antiangiogenic effect of sunitinib and DC101 was demonstrated by a significant reduction in CD31 staining in both ADC and SCC (Supplementary Fig S3). Central necrotic areas were identified in some lesions following antiangiogenic treatments (Supplementary Figs S4A,B). No bleeding episodes or fatal events were observed in mice treated with sunitinib or DC101.

**Figure 1:**
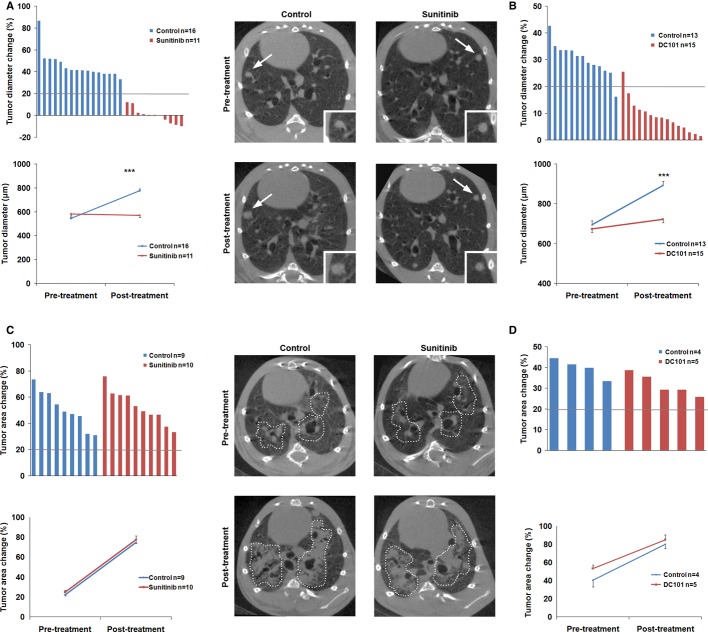
Sunitinib induces a differential tumor response between adenocarcinoma (ADC) and squamous cell carcinoma (SCC). A, B In the urethane-induced ADC mouse model, sunitinib (A) and DC101 (B) treatments stabilized tumor growth; Top: Waterfall plots of tumor response to sunitinib (A) or DC101 (B) treatments of ADC-bearing mice (the bold line at 20% indicates disease progression as defined by response in human cancers criteria). Tumor diameter change was calculated for each mouse taking the initial size of every lesion as reference. Each column represents the average tumor diameter change in each mouse after 5 weeks of treatment. Bottom: The pre-and post-treatment mean size for each group is presented in a line plot. Representative micro-CT images of ADC tumors show the progression of the disease in the control group and the stabilization of tumor growth in the sunitinib group. Lung ADC lesions (arrows) are shown at higher magnification (inset). Data are presented as mean ± standard error. C, D In the *N*-nitroso-tris-chloroethylurea-induced SCC model disease progression was observed. Top: Waterfall plots of SCC tumor response to sunitinib (C) or DC101 (D) treatments showing the percentage of area change for each mouse. Bottom: The pre-and post-treatment mean tumor area for each group is presented in a line plot. Representative micro-CT images of SCC tumors (dotted line) show the progression of the disease in the sunitinib and vehicle (control) groups. Data are presented as mean ± standard error.

Respiratory-gated micro-CT imaging was used to determine the effect of anti-VEGFR therapies on lung tumor progression. Longitudinal evaluation of treatment response was performed according to the criteria used for the assessment of response in human cancers (RECIST), using a threshold of 20% change in tumor diameter or tumor area as cutoff point to differentiate between progressive disease and stabilization of the disease (Eisenhauer *et al*, 2009). In ADC, tumor stabilization was achieved in mice treated with sunitinib (Fig 1A) or DC101 (Fig 1B). Conversely, mice bearing SCC tumors showed progression of the disease in sunitinib-(Fig 1C) and DC101-treated groups (Fig 1D). Mice receiving DC101 therapy earlier (8 weeks of NTCU treatment) also showed progression of the disease (Supplementary Fig S5). Therefore, in contrast to ADC, anti-VEGFR2 drugs failed to prevent tumor growth in the SCC mouse model.

### Inhibition of VEGFR2 differentially affects ADC and SCC tumor cell proliferation

**Figure 2:**
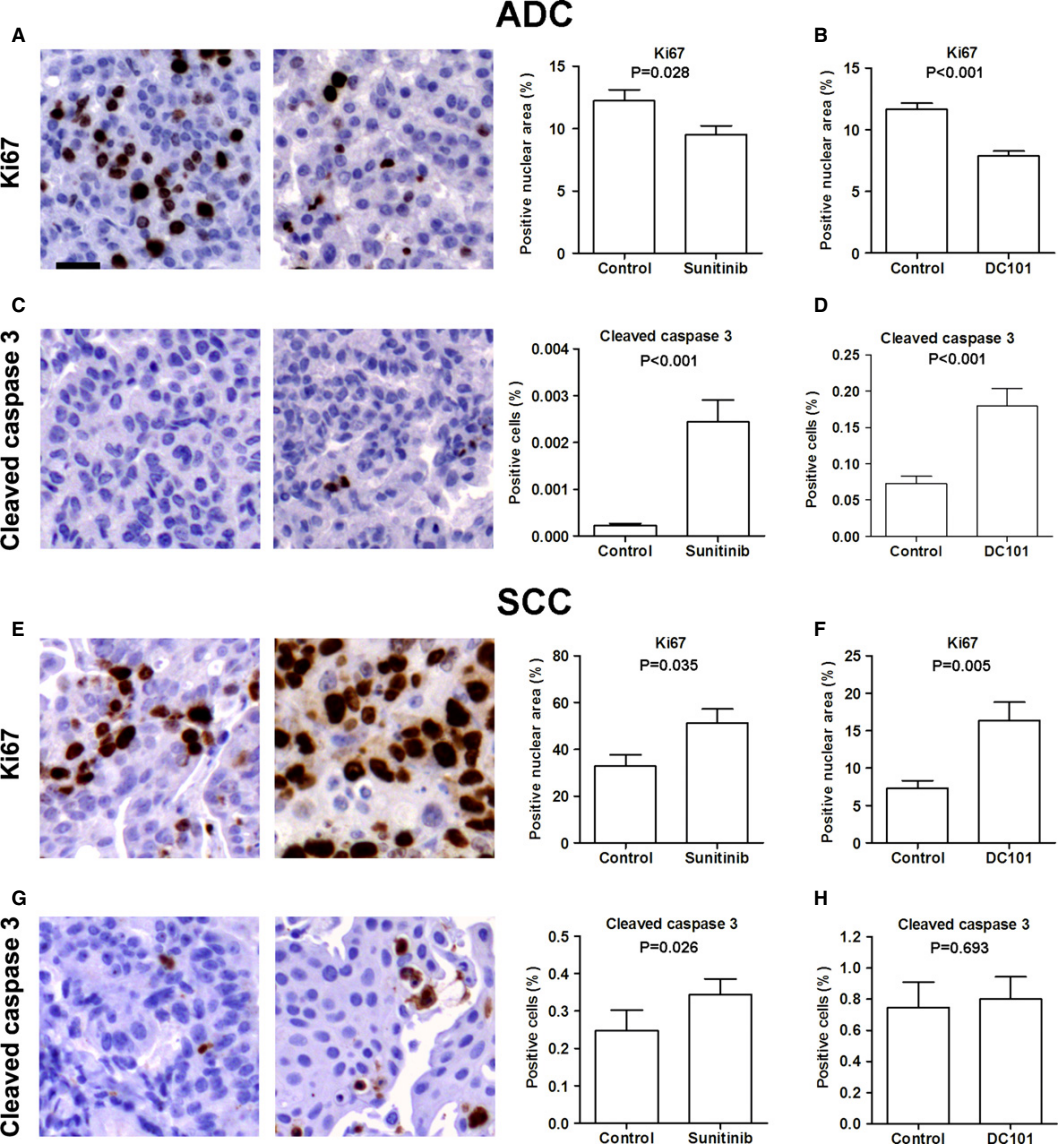
VEGFR2 blockade differentially affects adenocarcinoma (ADC) and squamous cell carcinoma (SCC) tumor cell proliferation and apoptosis. A–D Representative images of immunohistochemical analysis of Ki67 and cleaved caspase 3 in ADC tumor-bearing mice. In the urethane-induced ADC model, sunitinib (A) and DC101 (B) reduced the tumor cell proliferation index. Increased tumor apoptosis as measured by cleaved caspase 3 staining was observed in sunitinib (C) and DC101 (D) groups. Scale bar, 25 m. Data are presented as mean ± standard error. E–H Representative images of immunohistochemical analysis of Ki67 and cleaved caspase 3 in SCC. Sunitinib (E) and DC101 (F) treatments in SCC tumors significantly induced proliferation as compared to vehicle. The percentage of apoptotic cells was increased in the sunitinib group (G), but not in the DC101 group (H). Scale bar, 25 μm. Data are presented as mean ± standard error.

We next evaluated the effect of anti-VEGFR2 therapies on cell proliferation and apoptosis. In the ADC model, both sunitinib-and DC101-treated tumors showed a significant reduction in Ki67 staining when compared to their vehicle-treated counterparts (Figs 2A,B). Quantification of cleaved caspase 3 in ADC showed a significant increase in apoptosis in the sunitinib-(Fig 2C) and DC101-treated groups (Fig 2D). Contrastingly, in mice bearing lung SCC, sunitinib significantly increased tumor cell proliferation (Fig 2E). This increase in proliferation was also identified in DC101-treated animals (Fig 2F). The percentage of tumor-apoptotic cells also increased in the sunitinib group (Fig 2G), but not in the DC101 treatment (Fig 2H).

### The contrasting response to VEGFR2 inhibitors is also observed in ADC and SCC tumorgrafts

**Figure 3:**
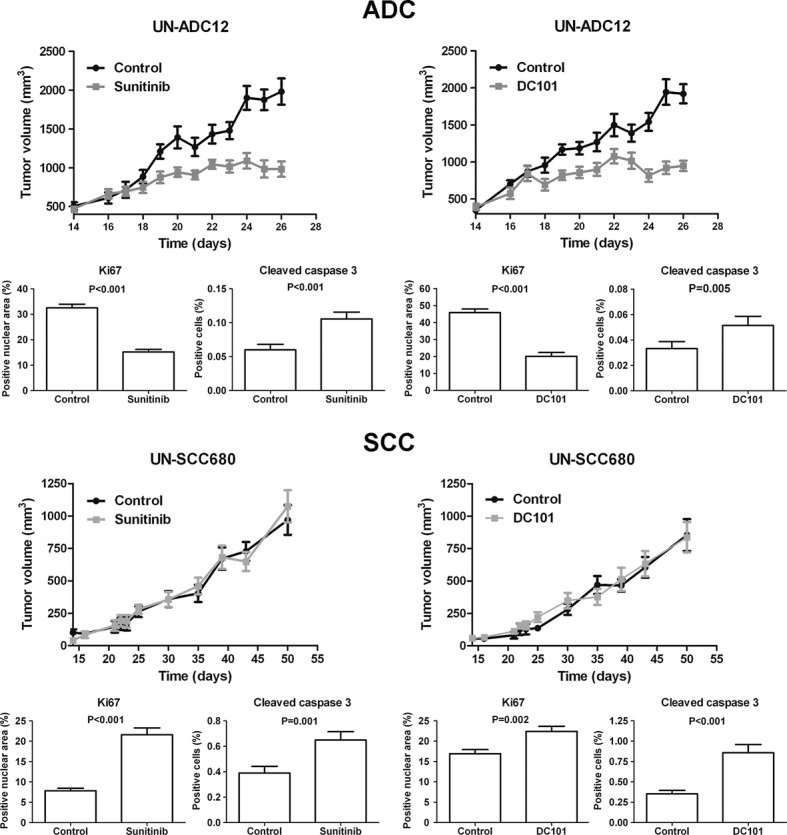
VEGFR2 inhibitors induce contrasting responses in adenocarcinoma (ADC) and squamous cell carcinoma (SCC) tumorgrafts. Immunodeficient mice bearing subcutaneous ADC or SCC tumors (using UN-ADC12 or UN-SCC680 cell lines, respectively) were treated with: sunitinib (40 mg/kg) or vehicle (PBS), or DC101 (40 mg/kg) or isotype antibody (40 mg/kg). Treatment was initiated at day 17 post-injection in the ADC model and at day 25 post-injection in the SCC model. The quantification of Ki67 and cleaved caspase 3 expression after sunitinib and DC101 treatments was performed automatically. Data are presented as mean ± standard error (*n* = 6 for all groups).

To further explore the contrasting efficacy of VEGFR2 inhibitors in ADC and SCC, subcutaneous implantation models were carried out with cell lines derived from urethane-induced tumors (UN-ADC12) and NTCU-induced tumors (UN-SCC680). Tumorgrafts bearing mice were treated with sunitinib or DC101. The antiangiogenic effect of the drugs was demonstrated by the evaluation of CD31 expression in tumorgrafts (Supplementary Fig S6). Tumor progression was evaluated by the assessment of changes in tumor volume longitudinally over time. UN-ADC12 tumorgrafts were significantly smaller in sunitinib-and DC101-treated groups than in the control group (Fig 3). In accordance with the results obtained in the urethane model, subsequent analysis of Ki67 and cleaved caspase 3 staining demonstrated that both sunitinib and DC101 treatments significantly reduced proliferation and increased apoptosis in ADC tumor cells (Fig 3). Moreover, histological analyses of the lungs, liver, spleen, and kidneys evidenced the presence of distant metastases (mainly to the lungs) that were significantly reduced with the anti-VEGFR2 treatments (Supplementary Fig S7). Overall survival was significantly prolonged in sunitinib-and DC101-treated mice as compared to control groups (Supplementary Figs S8A,B).

In mice bearing the SCC-derived UN-SCC680 tumorgrafts, no differences in tumor volume were found between sunitinib-or DC101-treated and control groups (Fig 3). Remarkably, both treatments significantly increased SCC tumor cell proliferation and apoptosis (Fig 3). These results confirm the hyperproliferative response observed in the *in vivo* NTCU model and suggest that a balance between proliferation and apoptosis in anti-VEGFR2-treated mice prevents tumor overgrowth as compared to controls. Moreover, no significant differences in overall survival were observed between groups (Supplementary Figs S8C,D). No distant metastases were found in this model.

### Anti-VEGFR2 treatments result in opposite survival and signaling effects in mouse ADC and SCC cell lines

**Figure 4:**
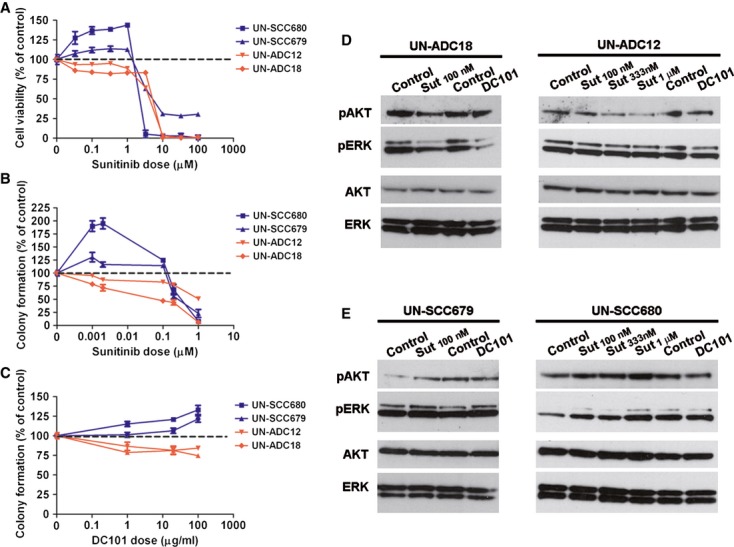
Anti-VEGFR2 therapies induce opposite effects on cell survival and VEGFR2 downstream signaling in *vitro*. A MTT cell proliferation assay of adenocarcinoma (ADC)-(UN-ADC12 and UN-ADC18) and squamous cell carcinoma (SCC)-(UN-SCC679 and UN-SCC680) tumor-derived cell lines treated with sunitinib. Data are presented as mean ± standard error. B, C Colony formation assay of ADC-and SCC tumor-derived cell lines treated with sunitinib (B) or DC101 (C). Data are presented as mean ± standard error. D, E Representative western blotting images of pAKT and pERK levels showing a differential activation of VEGFR2 downstream signaling in ADC (D) and SCC (E) cell lines. Source data are available for this figure.

To determine whether antiangiogenic treatments could directly affect cell survival independently of tumor microenvironment, we examined the effect of antiangiogenic drugs (sunitinib and DC101) on survival in cell lines derived from urethane-induced ADC (UN-ADC12 and UN-ADC18) and NTCU-induced SCC tumors (UN-SCC679 and UN-SCC680). In ADC cell lines, sunitinib treatment caused a modest inhibition of tumor cell proliferation (Fig 4A). However, sunitinib dramatically induced proliferation of SCC cell lines within the concentration range between 33.3 nM and 1 μM, whereas higher concentrations of sunitinib abolished cell proliferation. Those results were validated by cell survival assays that demonstrated the prosurvival effect of sunitinib and DC101 in SCC cell lines (Figs 4B,C). These results are in concordance with the *in vivo* experiments that demonstrated a higher tumor proliferative rate in SCC. We finally assessed the effect of VEGFR2 blockade on cell signaling. Consistent with the survival data presented above, sunitinib and DC101 treatments reduced the activation of AKT and ERK in ADC cell lines (Fig 4D). However, the phosphorylation levels of ERK and AKT were increased in SCC cell lines (Fig 4E) after sunitinib and DC101 treatments. Taken together, our results suggest that the opposite effects caused by the anti-VEGFR treatments in ADC and SCC tumor cells are associated with differences in signaling pathway activation.

### Antiangiogenic treatments increase the expression of stem cell markers in the SCC model

**Figure 5:**
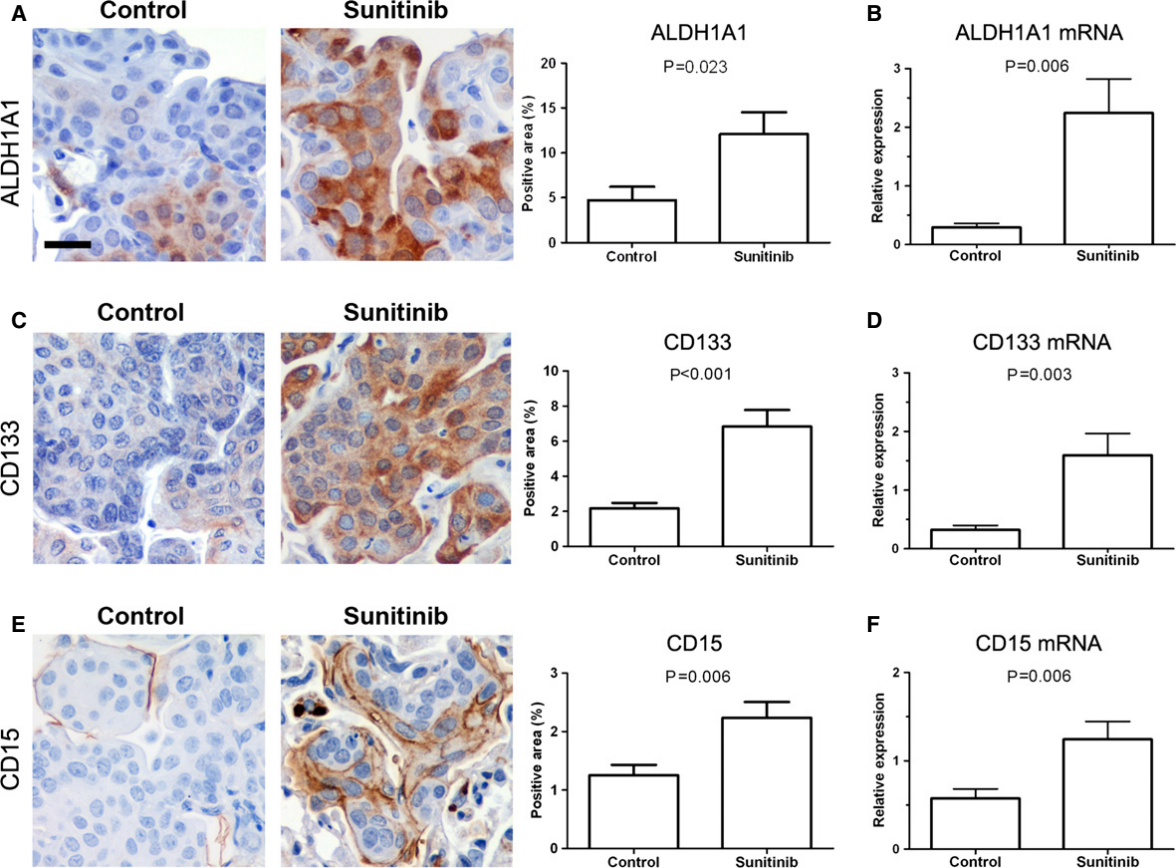
Sunitinib induces the expression of stem cell markers in squamous cell carcinoma tumors. A, B ALDH1A1 expression. Representative images of immunohistochemical analysis of ALDH1A1 in vehicle (control) and sunitinib-treated mice (A). Automatic quantification of tumor cell staining was performed. The mRNA expression of ALDH1A1 was evaluated by quantitative PCR (B). Each value was normalized to the reference gene (GUSB) and the expression in paired normal tissue was used for calibration. Scale bar, 25 μm. Data are presented as mean ± standard error. C, D CD133 expression analysis. Representative analysis of CD133 expression by immunohistochemistry (C) and by quantitative PCR (D). Each value was normalized to the reference gene (GUSB) and the expression in paired normal tissue was used for calibration. Scale bar, 25 m. Data are presented as mean ± standard error. E, F CD15 expression analysis by immunohistochemistry (E) and by quantitative PCR (F). Each value was normalized to the reference gene (GUSB) and the expression in paired normal tissue was used for calibration. Scale bar, 25 μm. Data are presented as mean ± standard error.

**Figure 6:**
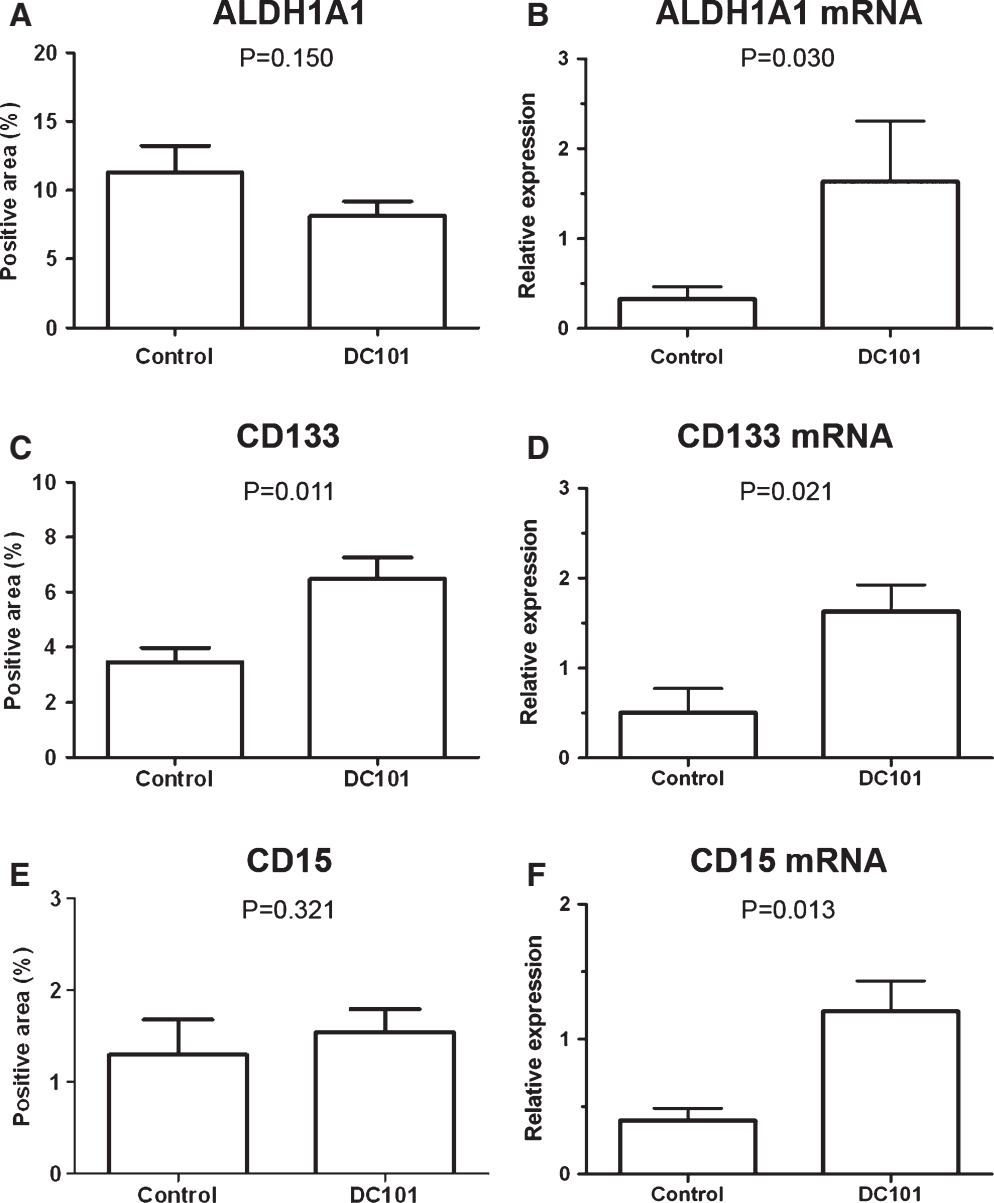
DC101 treatment induces the expression of stem cell markers in squamous cell carcinoma tumors. The expression of stem cell markers ALDH1A1, CD133, and CD15 was analyzed by immunohistochemistry (A, C, E) and quantitative PCR (qPCR) (B, D, F). Automatic quantification of tumor cell staining was performed. For qPCR, each value was normalized to the reference gene (GUSB) and the expression of paired normal tissue was used as calibrator sample. Data are presented as mean standard error. The expression of stem cell markers ALDH1A1, CD133, and CD15 was analyzed by immunohistochemistry (A, C, E) and quantitative PCR (qPCR) (B, D, F). Automatic quantification of tumor cell staining was performed. For qPCR, each value was normalized to the reference gene (GUSB) and the expression of paired normal tissue was used as calibrator sample. Data are presented as mean ± standard error. A, B ALDH1A1 expression. C, D CD133 expression E, F CD15 expression

Sunitinib increases the population of aldehyde dehydrogenase 1A1 (ALDH1A1)-positive cancer stem cells in breast cancer xenografts (Conley *et al*, 2012). In order to evaluate whether sunitinib increases this cell population in lung cancer, we analyzed the presence of ALDH1A1-positive cells in tumors from mice bearing urethane-induced ADC and NTCU-induced SCC. We also analyzed the expression of CD133 and CD15, other two markers that have been associated with stemness in lung cancer cells (Bertolini *et al*, 2009; Nolte *et al*, 2013). In the ADC model, stem cell markers expression was very low and no significant differences between groups were observed (Supplementary Fig S9A). In NTCU-induced SCC-bearing mice, tumor expression of ALDH1A1, CD133, and CD15 measured by immunohistochemistry was significantly increased following sunitinib treatment (Figs 5A,C,E). These results were further confirmed by real-time PCR (Figs 5B,D,F). After DC101 treatment, the mRNA expression of the three stem cell markers was significantly higher in DC101-treated SCC tumors, although only significant differences in CD133 were found when protein expression was evaluated (Fig 6). We next sought to evaluate the correlation between tumor hypoxia and stem cell markers, since hypoxia has been reported to be associated with the induction of ALDH1A1 and CD133 in some experimental models (Conley *et al*, 2012; Iida *et al*, 2012). Tumor hypoxia was measured by immunohistochemistry as the percentage of carbonic anhydrase IX (CA-IX)-positive cells and by real-time quantitative PCR (qPCR) as the expression of hypoxia-inducible factor-1α (HIF-1α). The expression of hypoxic markers was higher in mice treated with anti-VEGFR2 therapies (Supplementary Fig S10A), although differences were only statistically significant when CA-IX expression was analyzed. CA-IX expression did not correlate with ALDH1A1, CD133, or CD15 staining quantified in the same tumor areas of serial sections (Supplementary Figs S9B,C). Confirming this observation, no correlation between the expression of HIF-1α mRNA and ALDH1A1, CD133, or CD15 mRNA expression was found (Supplementary Fig S9D). Even when the correlation analyses were performed separately between control and treated mice (Supplementary Fig S10B), no correlation was found between hypoxia and stem cell markers. In conclusion, in the NTCU-induced SCC model, antiangiogenic treatments induce the expression of stem cell markers in a hypoxia-independent manner.

## Discussion

Antiangiogenic drugs targeting the VEGF signaling pathway have been approved in the treatment for NSCLC patients by means of successful clinical trials (Johnson *et al*, 2004; Sandler *et al*, 2006; Cohen *et al*, 2007; Reck *et al*, 2009). However, some limitations associated with antiangiogenic inhibitors have been reported (Ebos *et al*, 2009; Paez-Ribes *et al*, 2009; Casanovas, 2012; Conley *et al*, 2012; Van der Veldt *et al*, 2012). We recently showed that the prognostic relevance of the VEGF pathway in NSCLC is dependent on tumor histology (Pajares *et al*, 2012). Those results, together with the reported genetic differences between ADC and SCC histologies (Sos *et al*, 2009; Mountzios *et al*, 2010; Takahashi *et al*, 2010; Weiss *et al*, 2010; Hammerman *et al*, 2011, 2012; Imielinski *et al*, 2012; Sos & Thomas, 2012), led us to explore the efficacy of anti-VEGF/VEGFR therapy in clinically relevant mouse models that resemble the most represented NSCLC histologies. The choice of these models was based on the necessity to generate pure histological subtypes. Although there are several well-established genetically modified mouse models that resemble lung ADC tumors (Kwon & Berns, 2013), there are no validated transgenic mouse models that uniquely reproduce SCC tumors (Farago *et al*, 2012; You *et al*, 2013). In fact, the NTCU model is at present the only validated mouse model of lung SCC.

In mouse ADC tumors, we have demonstrated the therapeutic benefit of sunitinib and the monoclonal antibody DC101 against mouse VEGFR2. These results are in accordance with those reported in the *K-RAS* conditional mutant mouse model of lung ADC treated with sunitinib (Gandhi *et al*, 2009) and in the urethane mouse model treated with DC101 (Karoor *et al*, 2010). We have also demonstrated the therapeutic benefit of anti-VEGFR2 therapies in metastatic ADC tumorgrafts. VEGFR2 blockade was associated with a reduction in tumor cell proliferation and overall survival benefit. In contrast, we show for the first time that VEGFR2 blockade by sunitinib and DC101 leads to tumor progression in two experimental models of mouse SCC: a chemically induced mouse model of SCC and a model of SCC tumorgraft. Interestingly, VEGF pathway inhibition results in the hyperproliferation of SCC tumor cells in both models. This is supported by our *in vitro* observations that anti-VEGFR2 therapies induce cell proliferation and survival in SCC cell lines. These results demonstrate the relevance of the VEGF-VEGFR2 autocrine pathway in lung tumors, a circumstance that has been recently recognized in human cancers (Goel & Mercurio, 2013) and specifically demonstrated in human lung ADC cell lines (Chatterjee *et al*, 2013). More importantly, we demonstrate for the first time that anti-VEGFR therapies have protumoral effects on mouse models of lung SCC. In contrast, these therapies are able to control tumor progression in lung ADC models. Our study also suggests that differential cell signaling responses after VEGFR2 blockade could be one of the mechanisms underlying these contrasting differences on antitumor efficacy. In fact, although some reports demonstrate an association between VEGFR2 blockade and decrease in cell proliferation (Masood *et al*, 2001; Wu *et al*, 2007), in some ovarian and pancreatic cancer cell lines VEGFR2 knockdown enhances proliferation (Adham *et al*, 2010; Silva *et al*, 2011). Indeed, Chatterjee *et al* (2013)have reported that VEGFR2 knockdown in the EGFR-mutated H1975 human cell line of lung ADC is associated with higher proliferation and activation of ERK signaling in xenograft models. Interestingly, while urethane-induced ADC model is associated with K-RAS mutations (Fritz *et al*, 2010), this gene has not been found altered in NTCU-induced SCC tumors (Wang *et al*, 2004). Taken together, these data suggest that the specific mutation status underlying histological subtypes may be an important mediator of the response to antiangiogenic therapies. Furthermore, our data demonstrate that antiangiogenic drugs not only induce cell survival in SCC, but also increase the stem-like features of SCC tumors. Similar results were found in a breast cancer preclinical model after sunitinib treatment (Conley *et al*, 2012). The low expression of the three stem cell markers analyzed in this study in the urethane-induced ADC model reinforces the differences between ADC and SCC. Accordingly, heterogeneity of stem cell-related markers according to tumor subtype has been also described in other tumors (Park *et al*, 2010). In addition, it has been proposed that hypoxia may drive tumor growth through the expansion of cancer stem cells in severely hypoxic regions in tumors (Keith & Simon, 2007). In our model of SCC, antiangiogenic treatments induce the expression of stem cell markers independently of hypoxia. Our findings further link the induction of a stem cell population and the hyperproliferation of tumor with a lack of response to antiangiogenic drugs in SCC. These results, together with our previous observation in NSCLC patients that high VEGF pathway in early-stage SCC tumors correlates with good prognosis, emphasize the importance of a detailed preclinical evaluation of new antiangiogenic therapies. Moreover, our data support the need for precaution when considering the possibility of enrolling SCC patients in clinical trials evaluating antiangiogenic drugs.

## Materials and Methods

### Chemically induced animal models and treatment schedules

All animal experiments were conducted in accordance with the protocols approved by the Institutional Animal Care Committee.

For the chemically induced ADC model, A/J mice (Harlan Laboratories, Derby, UK) at 8 weeks of age received i.p. injection of urethane (Sigma-Aldrich, Madrid, Spain) in 0.9% saline at a dose of 1 g/kg body weight. Twelve weeks after injection, lung lesions were evaluated by respiratory-gated micro-CT analysis as previously described (Artaechevarria *et al*, 2010). For the SCC model, 8-week-old A/J mice were treated with 0.04 M *N*-nitroso-tris-chloroethylurea (NTCU; Toronto Research Chemicals, Inc., Toronto, ON, Canada) as previously described (Rehm *et al*, 1991; Wang *et al*, 2004). NTCU was applied by skin painting twice a week for 20 weeks.

Adenocarcinoma and SCC tumor-bearing mice were subjected to pre-treatment micro-CT analysis, randomly separated into four experimental groups, and treated over a time period of 5 weeks with the following scheme: (i) sunitinib malate (LC Laboratories, Woburn, MA, USA) at a dose of 40 mg/kg body weight was administered daily by oral gavage; (ii) oral gavage with phosphate-buffered saline (vehicle control) was applied to the control group for sunitinib; (iii) the monoclonal antibody against murine VEGFR2 (DC101; Bioxcell, West Lebanon, NH, USA) was i.p. injected at a dose of 40 mg/kg body weight twice a week; (iv) mice in the control group for DC101 were injected with a rat IgG1 antibody (Bioxcell) at 40 mg/kg body weight twice a week. Treatment schemes and doses of these drugs were chosen according to previous reports that demonstrated inhibition of VEGFR2 in animal models (Prewett *et al*, 1999; Christensen, 2007). For the SCC model, an additional DC101 treatment group in early SCC tumors was included. In brief, NTCU was applied for only 8 weeks and DC101 was injected for 5 weeks as previously described. Mice were subjected to an additional micro-CT to evaluate the response to treatment. Subsequently, mice were sacrificed and lungs were fixed in 4% buffered formalin for 24 h and paraffin-embedded.

Micro-CT images were calibrated to Hounsfield units using a water phantom. Tumor measurements (tumor diameter and tumor area) were performed by two independent observers using the ImageJ software (National Institutes of Health, Bethesda, MD, USA). Response evaluation criteria in human solid tumors (RECIST) were used to evaluate treatment response (Eisenhauer *et al*, 2009).

### Mouse cell line establishment

Two mouse SCC (UN-SCC679 and UN-SCC680) and two mouse ADC (UN-ADC12 and UN-ADC18) cell lines were established in our laboratory following the protocol previously published by Oie *et al* (1996) with minor modifications. Briefly, ADC tumors were induced by urethane injection and SCC tumors were induced by NTCU treatment, as described above. Lungs were excised after sacrifice and tumor cells were separated by the mechanical spillout method. Cells were cultured in ACL4 media (Oie *et al*, 1996) supplemented with 5% fetal bovine serum and penicillin–streptomycin–fungizone. Adherent colonies were cultured for at least 25 passages and subcutaneously injected in the flanks of 6-week-old female BALB/c Nu/Nu mutant athymic mice (UN-SCC679, UN-ADC12, and UN-ADC18 cell lines) or BALB/c-Rag2^−/−^-IL2γc^−/−^ immunodeficient mice (UN-SCC680). Tumorgrafts were excised as described above, and adherent colonies were cultured in ACL4 media.

### Subcutaneous tumorgraft assays

UN-SCC680 and UN-ADC12 cells (2 × 10^6^ cells) in an exponential growth phase were subcutaneously injected in the flanks of 5-to 7-week-old female BALB/c-Rag2^−/−^-IL2γc^−/−^ immunodeficient mice (*n* = 6 per experimental condition). Tumor size was measured externally using a precision caliper, and tumor volume (*V*) was calculated using the following equation: *V* = 0.50 × width^2^ × length. When tumors reached an average volume of 200 mm^3^ (UN-SCC680) or 700 mm^3^ (UN-ADC12), treatment was initiated. In brief, mice were treated with (i) 40 mg/kg sunitinib malate or (ii) PBS, both treatments daily oral gavage administered; (iii) 40 mg/kg DC101 or (iv) 40 mg/kg isotype antibody, both treatments administered by i.p. injection twice a week.

Experimental endpoint was accomplished when tumors reached 1.7 cm in diameter. Tumorgrafts, lungs, liver, spleen, and kidneys were harvested and fixed overnight in 4% buffered formalin, embedded in paraffin, and sectioned. The presence of tumor metastases was determined by hematoxylin and eosin (H/E) staining and histological examination. Lung H/E sections from the UN-ADC12 tumorgraft model were scanned, and tumor area was quantified using the ImageJ software.

### Cell proliferation and survival assays

MTT (Sigma-Aldrich) assay was used to evaluate cell proliferation. Cells were seeded at 1 × 10^3^ cells per well in 96-well plates and allowed to attach overnight. Cells were treated with PBS or sunitinib (at concentrations ranging from 33.3 nM to 100 μM) for 48 h, and cell proliferation was measured by MTT conversion. Absorbance was measured at 540/690 nm on a Sunrise plate reader (Tecan, Männedorf, Switzerland). The experiments were done in quintuplicate and repeated at least three times.

For clonogenic assays, cells were harvested and seeded in six-well plates (400 cells/well) and treated with sunitinib (1, 20, 100, 200 nM, or 1 μM), DC101 (1, 20, or 100 μg/ml), or vehicle: PBS for sunitinib control and rat IgG1 antibody (100 μg/ml) for DC101 control. After 14 days, cells were fixed in 4% buffered formalin for 30 min and stained with crystal violet to determine colony formation. All experiments were performed in triplicate and repeated at least three times.

### Western blotting

UN-ADC12, UN-ADC18, UN-SCC679, and UN-SCC680 cell lines were seeded at 300 000 cells per well in 6-well plates and allowed to attach overnight. Cells were treated with PBS, sunitinib (at concentrations ranging from 100 nM to 1 μM), DC101 (100 μg/ml), or rat IgG1 antibody (100 μg/ml) for 48 h. Cells were lysed and total proteins were extracted as previously described (Catena *et al*, 2010). Twenty μg of total protein from each lysate was boiled at 95°C for 5 min, separated by SDS–PAGE under reduced conditions (5% 2-mercaptoethanol), and transferred onto nitrocellulose membranes. The membranes were subsequently blocked in 5% defatted milk–PBS for 1 h and incubated overnight at 4°C with the following primary antibodies: anti-AKT (1:1000; 9272; Cell Signaling Technology, Beverly, MA, USA), anti-pAKT (1:1000; 9271; Cell Signaling Technology), anti-ERK (1:1000; 9102; Cell Signaling Technology), and anti-pERK (1:1000; 9101; Cell Signaling Technology). Blots were then incubated with a horseradish peroxidase-linked secondary antibody (1:2000; Amersham Pharmacia Biotech, Little Chalfont, UK) and developed by chemiluminescence with Lumilight plus kit (Roche diagnostics, Burgess Hill, UK).

### Immunohistochemistry

Immunohistochemistry of formalin-fixed, paraffin-embedded sections was performed using a Dako Autostainer (Dako, Madrid, Spain). Antibodies used for immunostaining were anti-VEGF (1:100; Sc-152 rabbit IgG; Santa Cruz Biotechnology, Heidelberg, Germany), anti-VEGFR2 (1:20; Sc-6251 mouse IgG1; Santa Cruz Biotechnology), anti-pVEGFR2 Tyr 1175 (1:20; 2478 rabbit IgG; Cell Signaling Technology), anti-TTF-1 (1:50; M3575 mouse IgG1; Dako), anti-p63 (1:50; N1604 mouse IgG2a; Dako), anti-CD31 (1:40; DIA310 rat IgG2a; Dianova, Hamburg, Germany), anti-Ki67 (1:100; SP6 rabbit IgG; Neomarkers, Fremont, CA, USA), anti-cleaved caspase 3 (1:100; 9661 rabbit IgG; Cell Signaling Technology), anti-ALDH1A1 (1:50; EP1933Y rabbit IgG; Abcam, Cambridge, UK), anti-CD133 (1:10; C24B9 rabbit IgG; Cell Signaling Technology), anti-CD15 (1:25; MC480 rabbit IgM; Abcam), and anti-CA-IX (1:00; ab15086 rabbit IgG; Abcam). For VEGF, VEGFR2, CD31, CD133, CD15, and CA-IX detection, microwave antigen retrieval was conducted with citrate buffer (10 mM, pH 6) for 20 min. For cleaved caspase 3 detection, microwave antigen retrieval with EDTA buffer (pH 8; Thermo Fisher Scientific, Waltham, MA, USA) was performed for 20 min. For phosphoVEGFR2, TTF-1, p63, Ki67, and ALDH1A1 detection, slides were incubated in Tris–EDTA buffer (pH 9; Thermo Fisher Scientific) for 20 min at 95°C in a Lab vision PT module (Thermo Fisher Scientific). After antigen retrieval, sections were incubated with the primary antibodies diluted in Real Antibody Diluent (Dako) for 30 min at room temperature. Subsequently, sections were incubated with the Envision complex (Dako), and the peroxidase activity was detected by 3,3′-diaminobenzidine. To detect CD31, a secondary antibody anti-rat immunoglobulins was applied before the Envision complex. Isotype control antibodies with no relevant specificity for lung tissue and obtained from the same species as the primary antibodies (Supplementary Fig S2B) or omission of the primary antibody were used as negative controls. Additional controls for VEGF pathway antibodies have been already published (Pajares *et al*, 2012).

**The paper explained**

**Problem**

The VEGF pathway is a clinically validated antiangiogenic target for advanced NSCLC. Hence, thousands of lung cancer patients are expected to be enrolled in clinical trials assessing the use of VEGF-pathway-targeted drugs. Since the prognostic relevance of the VEGF pathway in NSCLC patients is dependent on tumor histology, a rigorous assessment of the biological response to antiangiogenic drugs on the two main histologies of NSCLC is essential.

**Results**

Using mouse models of NSCLC-specific histological subtypes: ADC and SCC, we demonstrate here the contrasting responses of NSCLC to antiangiogenic therapies depending on histology. We have shown that two anti-VEGFR2 therapies (sunitinib and DC101) elicited vascular trimming and tumor stabilization in ADC tumors. In contrast, VEGFR2 blockade in SCC caused hyperproliferation of tumor cells and increased the expression of stem cell markers, independently of intratumoral hypoxia.

**Impact**

A major new finding of the present study is that antiangiogenic therapies induce contrasting responses in NSCLC depending on the histological subtype. Our findings further implicate the induction of stem cell population and tumor hyperproliferation as a mechanism for tumor progression in SCC treated with antiangiogenic drugs. These results have important clinical implications in the design of future antiangiogenic therapeutic trials and emphasize the need for precaution when considering the possibility of enrolling SCC patients in clinical trials evaluating antiangiogenic drugs.

Quantification of staining for CD31, Ki67, cleaved caspase 3, ALDH1A1, CD133, CD15, or CA-IX was performed automatically by the Analysis software (Olympus, Barcelona; Spain). Images were captured using a Zeiss Axio Imager M1 microscope (Carl Zeiss, Madrid, Spain). CD31-, ALDH1A1-, CD133-, CD15-, or CA-IX-positive area was calculated as the ratio of the positive area to total tumor area. In the case of Ki67, the ratio of positive nuclear area to total nuclear area was employed. For cleaved caspase 3 quantification, the ratio of positive cells to total tumor cells was calculated. In all cases, only tumor cell staining was quantified. In order to study the correlation between CA-IX and ALDH1A1, CD133, or CD15 expression, immunohistochemistry was performed on serial sections.

### Reverse transcription and real-time quantitative PCR

Total RNA was isolated from paired tumor and normal tissue on slides from paraffin-embedded sections (ten 3-μm-thick sections per mouse) using the RecoverAll Total Nucleic Acid Isolation system (Ambion, Life Technologies, Madrid, Spain). Five hundred nanograms of total RNA was reverse-transcribed to cDNA using SuperScript III (Invitrogen, Life Technologies, Madrid, Spain) and random hexamers (Applied Biosystems, Life Technologies, Madrid, Spain). Quantitative RT-PCR for ALDH1A1, CD133, CD15, and HIF-1α (primers sequences are listed in Supplementary Table S1) was performed using the SYBR Green kit (Applied Biosystems, Life Technologies) on an ABI PRISM 7300 real-time thermal cycler (Applied Biosystems, Life Technologies). All samples were run in triplicate and the genes of interest were normalized to the reference gene (GUSB) and the paired normal tissue using the 2^−ΔΔCt^ method.

### Statistical analysis

The normal distribution of the data was tested by the D'Agostino–Pearson test. Statistical differences between groups were evaluated by Student's *t* test or the Mann–Whitney *U* test according to data normality. Correlation analysis was performed by the Spearman rank test. Kaplan–Meier curves and the log-rank test were used to analyze differences in survival time. Differences were considered statistically significant when *P* values were <0.05. The statistical analysis was performed using SPSS v. 17.0 (SPSS Inc., Chicago, IL, USA) and GraphPad Prism v5.0 software (La Jolla, CA, USA).
