# Supplementary material for: Contrasting responses of non-small cell lung cancer to antiangiogenic therapies depend on histological subtype
Source: EMBO Mol Med. 2014 Feb 5;6(4):539–50. doi: 10.1002/emmm.201303214 (PMC3992079; doi:10.1002/emmm.201303214)
Supplement: Supplementary file 1 [file emmm0006-0539-sd1.pdf]

EMBO Molecular Medicine

Contrasting Responses of Non-Small Cell Lung Cancer to Antiangiogenic Therapies depend on histological subtype

Marta Larrayoz, Ruben Pio, María J. Pajares, Isabel Zudaire, Daniel Ajona, Oriol Casanovas, Luis M. Montuenga and Jackeline Agorreta

Corresponding author: Jackeline Agorreta, Center for Applied Medical Research-University of Navarra

Review timeline:

Submission date:	21 June 2013
Editorial Decision:	01 August 2013
Additional Author Correspondence:	02 August 2013
Revision received:	02 December 2013
Editorial Decision:	18 December 2013
Revision received:	27 December 2013
Accepted:	27 December 2013

Transaction Report:

(Note: With the exception of the correction of typographical or spelling errors that could be a source of ambiguity, letters and reports are not edited. The original formatting of letters and referee reports may not be reflected in this compilation.)

Editor: Roberto Buccione

1st Editorial Decision 01 August 2013

Thank you for the submission of your manuscript to EMBO Molecular Medicine. We are sorry that it has taken longer than usual to get back to you on your manuscript.

In this case we experienced unusual difficulties in securing three willing and appropriate reviewers. Further to this, we are still missing one evaluation. As a further delay cannot be justified, I have decided to proceed based on these available consistent evaluations. I should inform you that if in the meanwhile we should receive the third evaluation, but only if it raises significant caveats, these would need to be taken into consideration. We would not, however, ask you to comply with any further-reaching requests.

Reviewer 2 recognises the potential importance of the study but points to key confirmatory studies required to improve the manuscript. S/he notes that overall survival data are required to add critical insight to the effects of therapy and that details are lacking concerning the chemically-induced models used and why they were preferred over GEMMs. Reviewer 2 also wonders whether metastatic disease was also considered and why sunitinib- and DC101-treated SCC mice do not progress more than controls in SCC. This Reviewer also lists other very important experimental shortcomings and requests for clarification that require your action.

Reviewer 3 is also quite positive but would like you to provide some additional mechanistic insight and suggests some approaches to this effect. Although I will not be requiring you to perform these experiments (provided all other issues are carefully and fully dealt with), I do, however encourage

© EMBO

you to develop your study as far as realistically possible in a mechanistic sense for your next, revised version to strengthen your findings and increase their impact.

While publication of the paper cannot be considered at this stage, we would be prepared to consider a suitably revised submission, with the understanding that the Reviewers' concerns must be fully addressed with additional experimental data where appropriate and that acceptance of the manuscript will entail a second round of review.

Please note that it is EMBO Molecular Medicine policy to allow a single round of revision only and that, therefore, acceptance or rejection of the manuscript will depend on the completeness of your responses included in the next, final version of the manuscript.

As you know, EMBO Molecular Medicine has a "scooping protection" policy, whereby similar findings that are published by others during review or revision are not a criterion for rejection. However, I do ask you to get in touch with us after three months if you have not completed your revision, to update us on the status. Please also contact us as soon as possible if similar work is published elsewhere.

Should you find that the requested revisions are not feasible within the constraints outlined here and choose, therefore, to submit your paper elsewhere, we would welcome a message to this effect.

I look forward to seeing a revised form of your manuscript as soon as possible.

***** Reviewer's comments *****

Referee #2 (Remarks):

In the present study, Larrayoz et al. perform histological analysis to evaluate effects of VEGFR2 inhibitors, sunitinib and DC101, on two different types of non-small cell lung cancer. Using chemically induced lung carcinoma animal models for adenocarcinoma (ADC) and squamous-cell carcinoma (SSC), the authors demonstrate that treatment with both inhibitors induced vascular trimming in histological subtypes, but treatment-induced stabilization of the ADC tumors (observed by clinically relevant microCT evaluations) was not observed in SCC. The authors state this to be due to cell proliferation and survival induction, and increased stem-like features of this subtype of tumor. The data is well presented and the manuscript represents an important example of a preclinical study which follows clinically relevant parameters of progression and analysis. However, some key confirmatory studies are needed along with additional analysis before consideration for publication in EMBO Molecular Medicine.

Major points:

The authors present in vivo data from two different animal models, but more details on the pulmonary urethane-induced ADC and NTCU-induced SCC disease progression should be provided to clarify the role of VEGF pathway inhibition on disease progression. A stronger and clearer narrative with condensed figures would be helpful to highlight the main findings more succinctly. Some key outstanding questions the authors could address include:
1) If the authors microCT data shows that ADC treatment leads to a beneficial effect using the RECIST criteria, what is the effect on overall survival? Extended treatments would add critical insight into the overall effects of therapy in both models.
2) There are no details provided about the chemically induced models used in the text, nor is there justification why they are used instead of xenograft or genetically engineered mouse models. Does disease progression and morbidity include only primary disease, or do mice succumb to metastasis? This question is pertinent because a previous publication by Gandhi et al (cited by the authors) showed the effects of sunitinib in a genetically engineered mouse model of NSCLC can elicit beneficial effects in localized disease, but did not dramatically improve survival in a metastatic model. Moreover, there have been several recent publications that contrast not only the effects of VEGF pathway inhibitors in primary/localized versus metastatic disease, but also contrast the efficacies of antibody and TKI inhibition approaches. More data examining the effects of treatment are warranted to strengthen differential efficacies in SCC and ADC, perhaps with survival-based

studies, or inclusion of implantation models with established cell lines.
3) The authors demonstrate that sunitinib- and DC101-induce ki67 expression in the SCC models, but not the ADC model. This difference is suggested to be the primary reason for treatment inefficacy, and is linked to the observations that antiangiogenic therapy may enhance tumor growth. But why do sunitinib- and DC101-treated SCC mice not progress more than control animals in SCC?
4) There are no doses shown for figure 5 in the figure or a justification of why they were chosen. Instead, the fact that cells were exposed to 0.1 M sunitinib is mentioned only in the methods. Why was 0.1 M sunitinib chosen for an in vitro study? Clinically, there have been several papers demonstrating that the levels of circulating sunitinib in patients treated with the 4 week on/ 2 week off regimen is approximately 1 M, which is 10x the dose used in this study. Furthermore, several studies now demonstrate the levels of sunitinib that accumulate in the tumor (and skin) are far greater than what is present in the circulation - with levels reaching ~10 M (Gotink et al. Clin. Can. Res. 2011; Hammers et al. Mol. Can. Ther.). So the authors could be using concentrations 10-100x lower than clinically relevant levels. Perhaps compounding this problem further is that low doses of TKIs have been shown to 'stimulate' proliferation (Rose, et al. Brit. J. Pharm., 2010). With this in mind, it is suggested that the authors revise Figure 5 to include additional experiments. This could include IC50 studies with DC101 and sunitinib in their derived cell lines as well as the inclusion of established ADC and SCC cell lines. Also, since Ki67 is shown to increase after treatment, a proliferation assay should be performed to further examine the cell populations and an ADC cell line should be included.
6) In supplementary figure 4, the authors show that hypoxia is not correlated with stem cell markers. Using immunohistochemistry and quantitative PCR they show that hypoxia markers do not correlate with cancer stem cell markers. However they don't mention if the mice are control or treated mice. Since stem cell markers are increased after treatment a correlation should be made with treated mice compared to controls, where there are differences in hypoxia and stem cell marker expression. Furthermore, hypoxia should be quantified after each treatment.
7) The authors demonstrate that sunitinib and DC101 induce cell proliferation and increase stem cell markers in SCC, and suggest this as a reason for treatment inefficacy. But this link is not shown; ADC cells are not similarly examined; nor is this result tied to the potential that disease progression may actually be worse in certain tumor models and sub-types (mentioned in introduction). More in-depth study of this finding is recommended.

Minor points:

1) The authors must reorganize this paper so that the narrative follows the figure arrangement more carefully. As it stands, figures are referenced out of order, figures are not labeled, and the data seems more 'spread out' than it needs to be. The figures should be condensed for clarity. As an example, figure 1 really just recapitulates several previously published studies documenting VEGF pathway inhibition reduces CD31 - this could be shortened or moved to supplemental. Also, Figure 2 and Figure 3A-C make more sense together, and figure 3D-E and figure 4 are easier to understand side-by-side.
2) chemical induction started at the same age (8 weeks) but the differential tumor growth leads to differential treatment ignition timepoints (20 wks ADC; 28 wks SCC). Can age effects play a role in differential tumor response?
3) Figure 3B-C: without labels showing early and late treatments this experiment is very confusing.
4) In supplementary figure 2 the authors show the expression of VEGF, pVEGFR2 and tVEGFR2 in tumors. A negative control (IgG control) should be used to eliminate non-specific binding.
5) In supplementary figure 4 even though the authors state that immunohistochemistry was performed in serial tissue sections. It is hard to see evidence of this. Also, perhaps immunohistochemistry quantification, as well as p values and correlation lines in the correlation chart, could be added as well.

Referee #3 (Comments on Novelty/Model System):

The mouse NSCLC models involved in this study appear to be useful and appropriate for analysing

two different types of this disease - adenocarcinoma (ADC) and squamous cell carcinoma (SCC). Given this is the case, the medical impact and novelty of the findings based on these models is reasonable, but would be greater if some further mechanistic insight could be gained as to why ADC and SCC respond so differently to anti-angiogenic therapy.

Referee #3 (Remarks):

The finding that the response of different types of NSCLC mouse models to anti-angiogenic therapy is dependent on histology is of significance from a clinical perspective. But the manuscript would be of far greater interest if some mechanistic insight were demonstrated or at least suggested. To address this, the Authors should carefully explore if SCC tumor cells express higher levels of VEGFR-2 and/or co-receptors such as VEGFR-1 and the neuropilins, in vitro and in vivo, than ADC tumor cells. If so, this could be further explored in clinical samples. This issue needs to be addressed for the manuscript to have the appropriate scope.

Additional Author Correspondence 02 August 2013

We would like to thank the revision of our manuscript, entitled "Contrasting Responses of Non-Small Cell Lung Cancer to Antiangiogenic Therapies Depending on Histology". We are glad that the manuscript is potentially interesting for publication in EMBO Molecular Medicine, and will send a revised version of the manuscript in the next 2-3 months to be considered again.
Concerning the overall survival experiments pointed by reviewer 2, we will choose the alternative option suggested by the reviewer ("inclusion of implantation models with established cell lines") since the development of chemically induced models of overall survival would take more than a year of work. We think that the rest of the experiments suggested are feasible and reasonable, and we will perform them within the 3 months period.

Thank you very much for your kind consideration,

1st Revision - authors' response 02 December 2013

Responses to reviewers:

The authors would like to thank the reviewers for their insightful comments and suggestions. We have carefully revised the manuscript according to the reviewers' comments and have provided point-by-point responses. Changes in the manuscript text are underlined.

****** Reviewer's comments ******

Referee #2 (Remarks):

In the present study, Larrayoz et al. perform histological analysis to evaluate effects of VEGFR2 inhibitors, sunitinib and DC101, on two different types of non-small cell lung cancer. Using chemically induced lung carcinoma animal models for adenocarcinoma (ADC) and squamous-cell carcinoma (SSC), the authors demonstrate that treatment with both inhibitors induced vascular trimming in histological subtypes, but treatment-induced stabilization of the ADC tumours (observed by clinically relevant microCT evaluations) was not observed in SCC. The authors state this to be due to cell proliferation and survival induction, and increased stem-like features of this subtype of tumour. The data is well presented and the manuscript represents an important example of a preclinical study which follows clinically relevant parameters of progression and analysis. However, some key confirmatory studies are needed along with additional analysis before consideration for publication in EMBO Molecular Medicine.

Authors: We strongly appreciate your constructive review and the suggestions made for the improvement of our work. These are our replies to your comments:

Major points:

The authors present in vivo data from two different animal models, but more details on the pulmonary urethane-induced ADC and NTCU-induced SCC disease progression should be provided to clarify the role of VEGF pathway inhibition on disease progression. A stronger and clearer narrative with condensed figures would be helpful to highlight the main findings more succinctly. Some key outstanding questions the authors could address include:

1) If the authors microCT data shows that ADC treatment leads to a beneficial effect using the RECIST criteria, what is the effect on overall survival? Extended treatments would add critical insight into the overall effects of therapy in both models.

There are no details provided about the chemically induced models used in the text, nor is there justification why they are used instead of xenograft or genetically engineered mouse models. Does disease progression and morbidity include only primary disease, or do mice succumb to metastasis? This question is pertinent because a previous publication by Gandhi et al (cited by the authors) showed the effects of sunitinib in a genetically engineered mouse model of NSCLC can elicit beneficial effects in localized disease, but did not dramatically improve survival in a metastatic model. Moreover, there have been several recent publications that contrast not only the effects of VEGF pathway inhibitors in primary/localized versus metastatic disease, but also contrast the efficacies of antibody and TKI inhibition approaches. More data examining the effects of treatment are warranted to strengthen differential efficacies in SCC and ADC, perhaps with survival-based studies, or inclusion of implantation models with established cell lines.

Author´s response: The choice of chemically induced murine models was based on the necessity of obtaining mouse models of pure histological subtype. Although there are several well established genetically modified mouse models that resemble lung ADC tumors (Kwon & Berns, 2013), there are no validated transgenic mouse models that uniquely reproduce SCC tumors (Farago et al, 2012; You et al, 2013). In fact, the NTCU model is currently the only validated mouse model of lung SCC.

The reason for choosing chemically-induced models rather than genetically engineered mouse models is now appropriately explained in the manuscript (page 10).

Unfortunately, as it is usual in almost every lung cancer chemically induced model, metastases cannot be found in either urethane or NTCU treated mice. Therefore, following the reviewer's suggestion, we have performed survival-based studies using subcutaneous implantation models of mouse ADC and SCC with established cell lines. As expected, these studies clearly confirm our previously observed response in ADC to antiangiogenic treatments. More importantly, we have also validated the incapacity of VEGFR2 blockade to control tumor progression in SCC-grafted tumors and its induction of proliferation of SCC tumor cells. We have also shown that sunitinib and DC101 treatments reduce microvascular density and induce apoptosis in both ADC and SCC tumor graft models. All these data have been added to the Results section and are shown in Figure 3 and Supplementary Figures 6-8. We appreciate the reviewer's suggestion, because this new information will make the paper more robust.

3) The authors demonstrate that sunitinib- and DC101-induce ki67 expression in the SCC models, but not the ADC model. This difference is suggested to be the primary reason for treatment inefficacy, and is linked to the observations that antiangiogenic therapy may enhance tumour growth. But why do sunitinib- and DC101-treated SCC mice not progress more than control animals in SCC?

Author´s response: Tumor growth is regulated by the balance between proliferation and cell death. Both in ADC and SCC models antiangiogenic agents have a deep impact increasing the percentage of apoptotic cells. The final effect of low proliferation and high apoptosis in ADC cells is correlated with a reduction of tumor volume. However, in SCC both proliferation and apoptosis pathways are activated, so we may argue that this is the reason why sunitinib- and DC101- treated mice do not progress more than control animals. A comment on this hypothesis has been added in the revised version (page 7).

4) There are no doses shown for figure 5 in the figure or a justification of why they were chosen. Instead, the fact that cells were exposed to 0.1μM sunitinib is mentioned only in the methods. Why was 0.1μM sunitinib chosen for an in vitro study? Clinically, there have

been several papers demonstrating that the levels of circulating sunitinib in patients treated with the 4 week on/ 2 week off regimen is approximately 1μM, which is 10x the dose used in this study. Furthermore, several studies now demonstrate the levels of sunitinib that accumulate in the tumour (and skin) are far greater than what is present in the circulation - with levels reaching ~10μM (Gotink et al. Clin. Can. Res. 2011; Hammers et al. Mol. Can. Ther.). So the authors could be using concentrations 10-100x lower than clinically relevant levels. Perhaps compounding this problem further is that low doses of TKIs have been shown to 'stimulate' proliferation (Rose, et al. Brit. J. Pharm.,2010). With this in mind, it is suggested that the authors revise Figure 5 to include additional experiments. This could include IC50 studies with DC101 and sunitinib in their derived cell lines as well as the inclusion of established ADC and SCC cell lines. Also, since Ki67 is shown to increase after treatment, a proliferation assay should be performed to further examine the cell populations and an ADC cell line should be included.

Author´s response: As the reviewer pointed out, we had performed clonogenic assays with SCC cell lines and only one dose for each treatment: sunitinib (0.1 μM) and DC101 (100 μg/ml). According to the suggestion of the reviewer, we have now performed new experiments that include proliferation studies (MTT assay) with a dose range of sunitinib (33.3 nM to 100 μM) both in ADC and SCC cell lines. Moreover, we have performed clonogenic assays with ADC and SCC cell lines treated with sunitinib (1 nM to 1 μM) and DC101 (1 to 100 μg/ml). We have analyzed in these experiments two new ADC cell lines (UN-ADC12 and UN-ADC18) derived from urethane-induced ADC tumors. In ADC cell lines, sunitinib and DC101 treatments inhibited both proliferation and cell survival in a dose-dependent manner. However, VEGR2 blockade induced proliferation and cell survival in SCC cell lines at concentrations ranging between 33.3 nM and 1 μM in the case of MTT assay and between 0.1 and 100 nM in clonogenic assays. It should be noted that the differences between the effective range of concentrations between MTT and clonogenic assays is due to the intrinsic characteristics of both assays. While MTT evaluates short term proliferation of tumor cells (48 h after treatment initiation), clonogenic assay analyzes long term cell survival. These results demonstrate that antiangiogenic treatments elicit contrasting effects on proliferation and cell survival on ADC and SCC cell lines. We strongly believe that these results are clinically relevant and confirm the results obtained in the “in vivo” models. We have added the MTT and clonogenic assays results as new Figure 4, and referred to them in the Results section (pages 6 and 7).

6) In supplementary figure 4, the authors show that hypoxia is not correlated with stem cell markers. Using immunohistochemistry and quantitative PCR they show that hypoxia markers do not correlate with cancer stem cell markers. However they don't mention if the mice are control or treated mice. Since stem cell markers are increased after treatment a correlation should be made with treated mice compared to controls, where there are differences in hypoxia and stem cell marker expression. Furthermore, hypoxia should be quantified after each treatment.

Author´s response: We thank the reviewer for this suggestion. We now provide more detailed information about the treatment of each mouse (Supplementary Figure 9) and we also report the value of immunohistochemical expression of each marker (Supplementary Figure 9). As suggested by the reviewer, we have performed a separate analysis of correlation in control and treated mice and compared hypoxic marker expression in control and treated mice. We concluded that there is no correlation between hypoxia and stem cell markers both in control and treated groups, even were analyzed separately. These results are now presented in Supplementary Figure 10.

7) The authors demonstrate that sunitinib and DC101 induce cell proliferation and increase stem cell markers in SCC, and suggest this as a reason for treatment inefficacy. But this link is not shown; ADC cells are not similarly examined; nor is this result tied to the potential that disease progression may actually be worse in certain tumour models and sub-types (mentioned in introduction). More in-depth study of this finding is recommended.

Author´s response: In the present study we have demonstrated the inefficacy of antiangiogenic treatments using “in vivo” models of mouse lung SCC. The hyperproliferative response of SCC tumor cells to anti-VEGFR2 drugs observed “in vitro” and “in vivo” points to a detrimental effect of these therapies in the SCC models studied herein. Moreover, we have determined that VEGFR2 blockade induced the expression of stem cell markers in SCC. This observation has been related to the lack of effect of antiangiogenic treatments in other tumors such as breast cancer (Conley et al, 2012). We agree with the reviewer that our results demonstrate the association between stem cells

and treatment inefficacy more than a direct link. Therefore, we have to be cautious in our conclusions. Nevertheless, it could be argued that the increase of the stem cell population by therapeutic agents may increase the self-renewal potential and the ability to initiate the growth of new tumors. We agree with the reviewer that the study of stem cell markers expression in ADC model was necessary to do the appropriate comparison between ADC and SCC models. Accordingly, we have now included new results providing this information in the Supplementary Figure 9A. We found that the expression of the three stem cell markers was very low in ADC, and no differences were found after antiangiogenic treatments. These results reinforce the differences between ADC and SCC.

As mentioned by the reviewer, there may be potential differences of disease progression between the experimental models reported in the present manuscript. Since we were aware of this circumstance, we included vehicle-treated control groups in the experimental design of each "in vivo" model.

Minor points:

1) The authors must reorganize this paper so that the narrative follows the figure arrangement more carefully. As it stands, figures are referenced out of order, figures are not labelled, and the data seems more 'spread out' than it needs to be. The figures should be condensed for clarity. As an example, figure 1 really just recapitulates several previously published studies documenting VEGF pathway inhibition reduces CD31 - this could be shortened or moved to supplemental. Also, Figure 2 and Figure 3A-C make more sense together, and figure 3D-E and figure 4 are easier to understand side-by-side.

Author´s response: According to referee´s suggestions, we have reorganized figures and labels. In particular, Figure 1 is now a supplementary figure and Figures 2 and 3 have been restructured.

2) Chemical induction started at the same age (8 weeks) but the differential tumour growth leads to differential treatment ignition time points (20 wks ADC; 28 wks SCC). Can age effects play a role in differential tumour response?

Author´s response: As the reviewer pointed out, the necessary time to develop tumours is different in urethane and NTCU models; therefore, the age of animals at the end of the treatments differs between both models. Nevertheless, we deliberately decided to initiate tumor induction at the same time point in order to guarantee homogenous immune system development in both models. In each model, tumor growth in sunitinib and DC101 groups was compared to their respective age-matched control groups. Moreover, in SCC DC101 therapy was also evaluated at an early stage of the disease (8 weeks of NTCU treatment; 21 weeks at the end of the experiment) and the same results were found. Therefore, we believe that age is not playing an essential role in the differential responses between ADC and SCC mouse models.

3) Figure 3B-C: without labels showing early and late treatments this experiment is very confusing.

Author´s response: We have reorganized figures according to reviewer´s suggestion, and early DC101 treatment has been moved to supplemental (see Supplemental Figure 5).

4) In supplementary figure 2 the authors show the expression of VEGF, pVEGFR2 and tVEGFR2 in tumours. A negative control (IgG control) should be used to eliminate non-specific binding.

Author´s response: According to the suggestion of the reviewer, we provide a picture of the isotype control for all the antibodies used in the present study (See Supplemental Figure 2B).

5) In supplementary figure 4 even though the authors state that immunohistochemistry was performed in serial tissue sections. It is hard to see evidence of this. Also, perhaps immunohistochemistry quantification, as well as p values and correlation lines in the correlation chart, could be added as well.

Author´s response: As suggested by the reviewer, we added information regarding the immunohistochemistry quantification chart for each tumor (Supplemental Figure 9C).

Referee #3 (Comments on Novelty/Model System):

The mouse NSCLC models involved in this study appear to be useful and appropriate for analysing two different types of this disease - adenocarcinoma (ADC) and squamous cell carcinoma (SCC). Given this is the case, the medical impact and novelty of the findings based on these models is reasonable, but would be greater if some further mechanistic insight could be gained as to why ADC and SCC respond so differently to anti-angiogenic therapy.

Thank you for evaluating our results and for your encouraging comments. These are our replies to your comments:

Referee #3 (Remarks):

The finding that the response of different types of NSCLC mouse models to anti-angiogenic therapy is dependent on histology is of significance from a clinical perspective. But the manuscript would be of far greater interest if some mechanistic insight were demonstrated or at least suggested. To address this, the Authors should carefully explore if SCC tumour cells express higher levels of VEGFR-2 and/or co-receptors such as VEGFR-1 and the neuropilins, in vitro and in vivo, than ADC tumour cells. If so, this could be further explored in clinical samples. This issue needs to be addressed for the manuscript to have the appropriate scope.

Author´s response: There are several potential underlying biological mechanisms which could explain the differences between ADC and SCC showed in the present manuscript. We agree with the reviewer that the differential expression of the molecules implied in the VEGF/VEGFR pathway between ADC and SCC tumours may have an impact in the efficacy of anti-VEGFR2 agents. In fact, we have previously reported in human NSCLC samples that the expression of VEGF, VEGFR1 and VEGF is significantly higher in ADC than in SCC tumour samples (Pajares et al, 2012). In order to provide additional mechanistic insights, new "in vitro" experiments with ADC and SCC cell lines derived from urethane and NTCU induced tumours have now been performed and included in the manuscript (Figure 4). We have included in these experiments two new ADC cell lines (UN-ADC12 and UN-ADC18) derived from urethane-induced ADC tumors, together with the previously reported SCC cell lines (UN-SCC679 and UN-SCC680). ADC and SCC cell lines were treated with PBS, sunitinib, DC101 or isotype control and the effect of VEGFR2 blockade on cell signaling was assessed by studying the activation of AKT and ERK by immunoblotting analysis. Consistent with our previous results, sunitinib and DC101 treatments inhibited AKT and ERK phosphorylation in ADC cell lines. Remarkably, pERK and pAKT signaling was increased in SCC cell lines in a dose-dependent manner. It could be reasoned that the activation of the AKT and ERK signaling pathway in SCC cell lines after VEGFR2 blockade has an impact on the induction of proliferation. Moreover, it is important to notice that while urethane-induced ADC model is associated with K-RAS mutations (Fritz et al, 2010), this gene has not been found altered in NTCU-induced SCC tumors (Wang et al, 2004). Indeed, it has been recently reported that VEGFR2 knockdown is associated with higher proliferation in the EGFR mutated H1975 human cell line (Chatterjee et al, 2013). Taken together, these data suggest that the specific mutation status underlying histological subtypes may be an important mediator of the response to antiangiogenic therapies. Some sentences summarizing these arguments in the discussion section of the manuscript (page 11).

We appreciate the reviewer's comments, which helped us to improve the quality of the paper.

References:

Conley SJ, Gheordunescu E, Kakarala P, Newman B, Korkaya H, Heath AN, Clouthier SG, Wicha MS (2012) Antiangiogenic agents increase breast cancer stem cells via the generation of tumor hypoxia. Proc Natl Acad Sci U S A 109: 2784-2789

Chatterjee S, Heukamp LC, Siobal M, Schottle J, Wieczorek C, Peifer M, Frasca D, Koker M, Konig K, Meder L et al (2013) Tumor VEGF:VEGFR2 autocrine feed-forward loop triggers angiogenesis in lung cancer. J Clin Invest 123: 1732-1740

Farago AF, Snyder EL, Jacks T (2012) SnapShot: Lung cancer models. Cell 149: 246-246 e241

Fritz JM, Dwyer-Nield LD, Russell BM, Malkinson AM (2010) The Kras mutational spectra of chemically induced lung tumors in different inbred mice mimics the spectra of KRAS mutations in adenocarcinomas in smokers versus nonsmokers. J Thorac Oncol 5: 254-257

Kwon MC, Berns A (2013) Mouse models for lung cancer. Mol Oncol 7: 165-177

Pajares MJ, Agorreta J, Larrayoz M, Vesin A, Ezponda T, Zudaire I, Torre W, Lozano MD, Brambilla E, Brambilla C et al (2012) Expression of tumor-derived vascular endothelial growth factor and its receptors is associated with outcome in early squamous cell carcinoma of the lung. J Clin Oncol 30: 1129-1136

Wang Y, Zhang Z, Yan Y, Lemon WJ, LaRegina M, Morrison C, Lubet R, You M (2004) A chemically induced model for squamous cell carcinoma of the lung in mice: histopathology and strain susceptibility. Cancer Res 64: 1647-1654

You MS, Rouggly LC, You M, Wang Y (2013) Mouse models of lung squamous cell carcinomas. Cancer Metastasis Rev 32: 77-82

2nd Editorial Decision 18 December 2013

Thank you for the submission of your revised manuscript to EMBO Molecular Medicine. We have now received the enclosed reports from the referees that were asked to re-assess it. As you will see the reviewers are now supportive and I am pleased to inform you that we will be able to accept your manuscript pending the following final amendments:

1) As you will see, Reviewer 2 would like you to amend and clarify an interpretation issue with Supplemental Figure 8. Please do so and also provide an additional copy of your manuscript highlighting the changes in the main text. Your amendment will be evaluated at the Editorial level.

2) We would need a short list (up to 5) of bullet points that summarize the key NEW findings. The bullet points should be in the third person and designed to be complementary to the abstract and will be used online in our new platform (coming January 2014).

3) I would like to try to make the title a bit more impactful. For instance, what do you think of the following alternatives "Contrasting Responses of Non-Small Cell Lung Cancer to Antiangiogenic Therapies depend on histological subtype" or "Histological subtype predicts Non-Small Cell Lung Cancer response to Antiangiogenic Therapies"? Would you care to suggest an alternative(s) yourself?

4) Finally, we are now encouraging the publication of source data, particularly for electrophoretic gels and blots, with the aim of making primary data more accessible and transparent to the reader. Would you be willing to provide a PDF file per figure that contains the original, uncropped and unprocessed scans of all or at least the key gels used in the manuscript? The PDF files should be

labeled with the appropriate figure/panel number, and should have molecular weight markers; further annotation may be useful but is not essential. The PDF files will be published online with the article as supplementary "Source Data" files. If you have any questions regarding this just contact me.

Please submit your revised manuscript within two weeks. I look forward to seeing a revised form of your manuscript as soon as possible.

***** Reviewer's comments *****

Referee #2 (Remarks):

In the revised submission, Larravoz et al have adequately addressed the concerns raised in our initial review and we recommend publication. We have one correction suggested (see below).

Comment:

In Supplemental Figure 8, the authors have added data from tumor xenograft studies to show the differences in treatment effects on overall survival. However, these studies appear to be only ectopically grown tumors and 'survival' is when a tumor reaches institutional limits (1.7 cm). if this is correct, then we disagree with this being described as a 'survival' study as these mice have localized disease, and does not represent clinically relevant systemic metastatic disease endpoints. We therefore recommend changing the axis title to something more accurate, such as '% reaching endpoint', with a reference in the legend describing what this endpoint is.

Referee #3 (Remarks):

The Authors have satisfactorily addressed my concern regarding the scope of the manuscript.

2nd Revision - authors' response 27 December 2013

Responses to reviewers:

Referee #2 (Remarks):

In the revised submission, Larravoz et al have adequately addressed the concerns raised in our initial review and we recommend publication. We have one correction suggested (see below).

Comment:

In Supplemental Figure 8, the authors have added data from tumour xenograft studies to show the differences in treatment effects on overall survival. However, these studies appear to be only ectopically grown tumours and 'survival' is when a tumour reaches institutional limits (1.7 cm). if this is correct, then we disagree with this being described as a 'survival' study as these mice have localized disease, and does not represent clinically relevant systemic metastatic disease endpoints. We therefore recommend changing the axis title to something more accurate, such as '% reaching endpoint', with a reference in the legend describing what this endpoint is.

As suggested by reviewer 2, the legend of the supplementary Figure 8 has been modified and the experimental endpoint of the survival experiments has been clarified.

Referee #3 (Remarks):

The Authors have satisfactorily addressed my concern regarding the scope of the manuscript.
