# Supplementary figures and images for: Contrasting responses of non-small cell lung cancer to antiangiogenic therapies depend on histological subtype
Source: EMBO Mol Med. 2014 Feb 5;6(4):539–50. doi: 10.1002/emmm.201303214 (PMC3992079; doi:10.1002/emmm.201303214)

Western blot images figure 4

Figure 4D

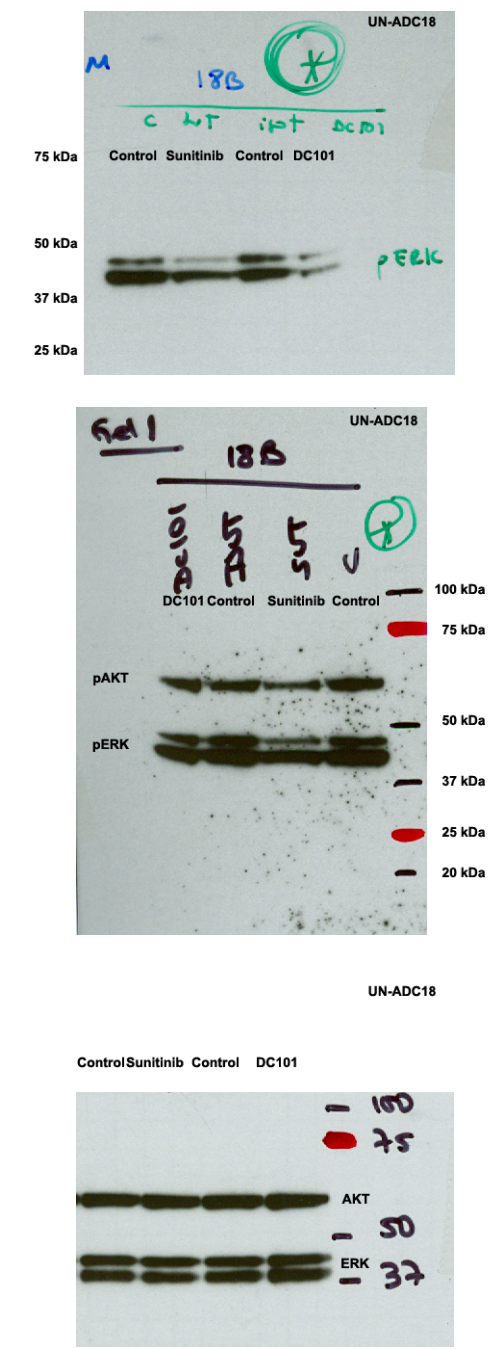

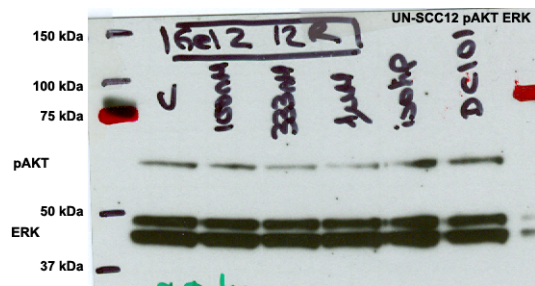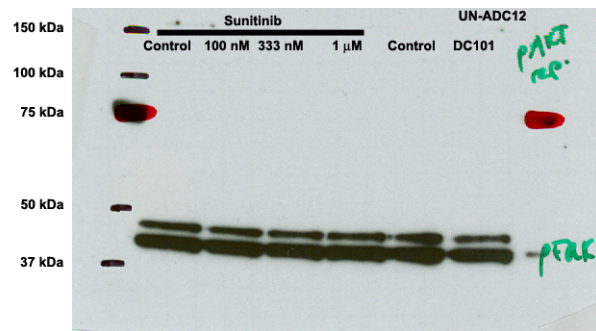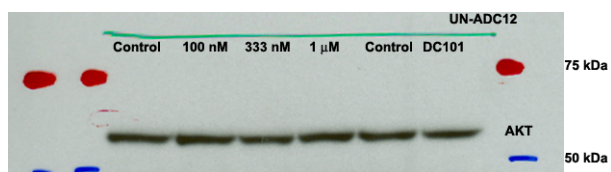

Supplement: Supplementary file 2 [file emmm0006-0539-sd2.pdf]

Figure 4E

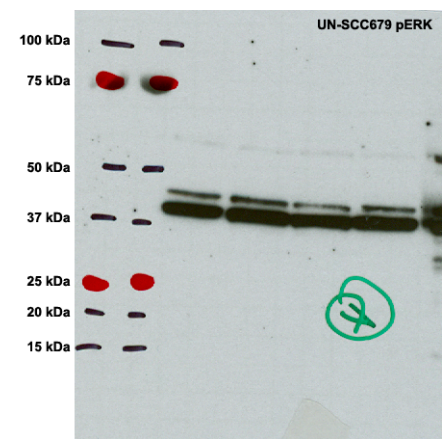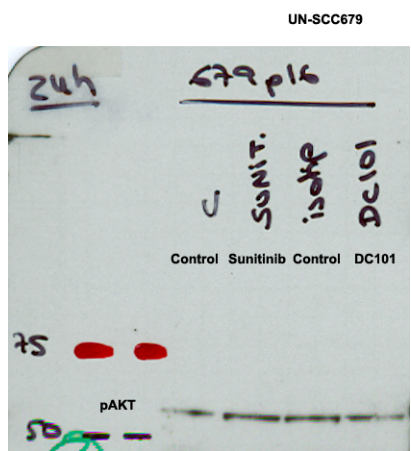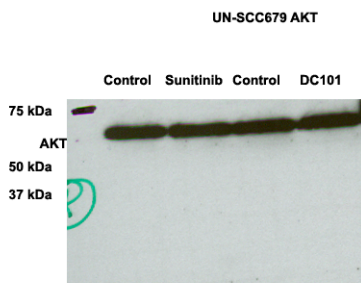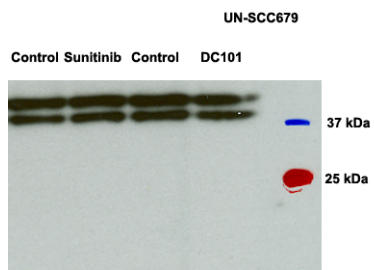

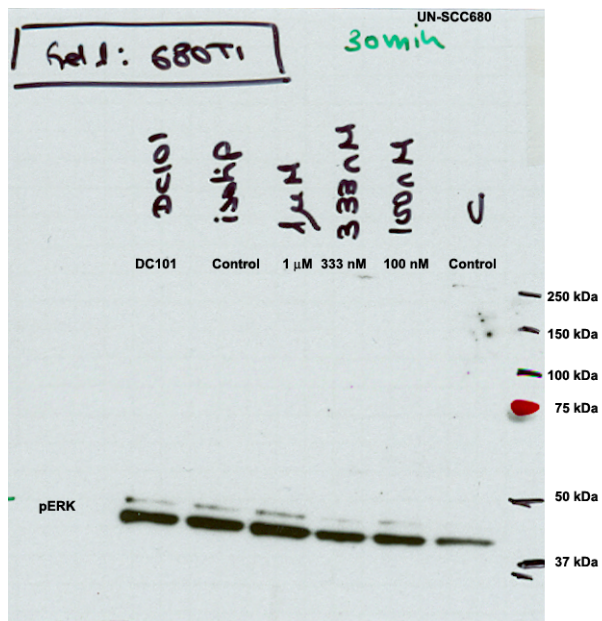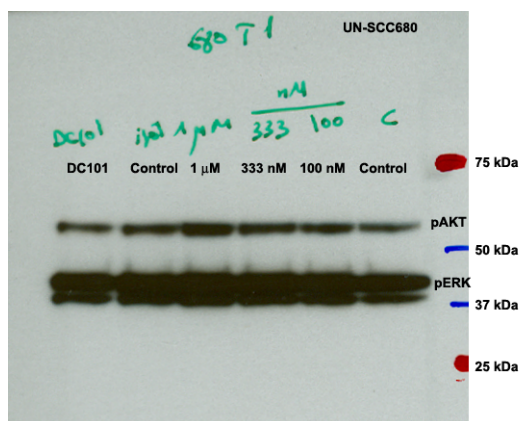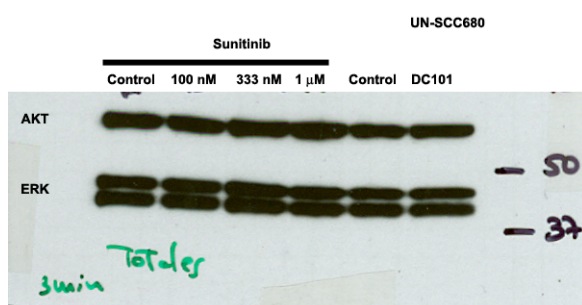

Supplement: Supplementary file 3 [file emmm0006-0539-sd3.pdf]
