# Supplementary material for: Contrasting responses of non-small cell lung cancer to antiangiogenic therapies depend on histological subtype
Source: EMBO Mol Med. 2014 Feb 5;6(4):539–50. doi: 10.1002/emmm.201303214 (PMC3992079; doi:10.1002/emmm.201303214)
Supplement: Supplementary file 4 [file emmm0006-0539-sd4.pdf]

SUPPLEMENTARY FIGURES

Supplementary Figure 1

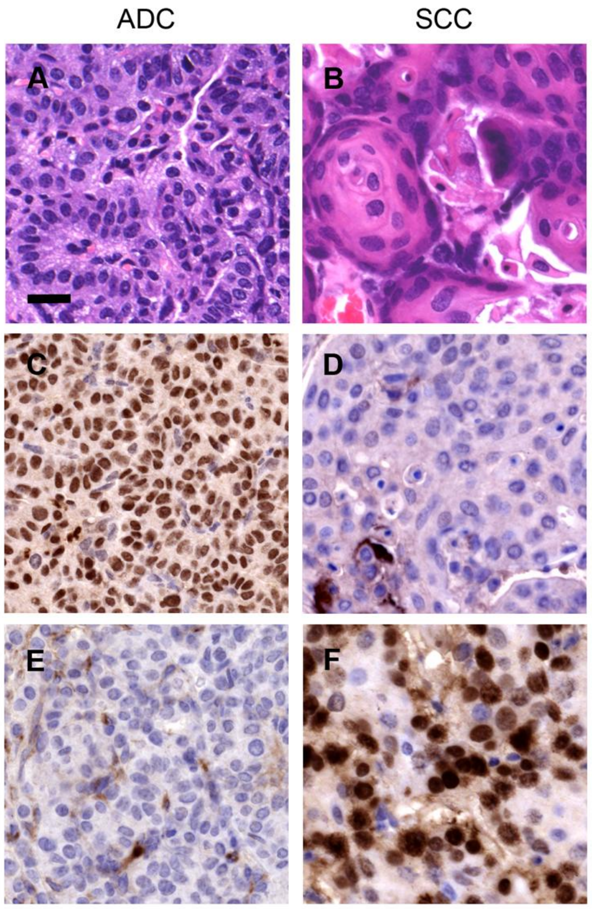

Supplementary Figure 1. Histological evaluation of chemically-induced murine lung tumors. (A) H&E staining of a nodule from the urethane model showing a glandular growth pattern. (B) H&E of a NTCU tumor. Tumor cells exhibited squamous differentiation, with keratin pearl formation. The characterization of the lesions was performed by immunohistochemistry for TTF1 (C, D) and p63 (E, F). Urethane tumors were positive for TTF1 (C) and negative for p63 (E) whereas NTCU tumors were negative for TTF1 (D) and positive for p63 (F). Scale bar, 25 μm.
