# Supplementary material for: Contrasting responses of non-small cell lung cancer to antiangiogenic therapies depend on histological subtype
Source: EMBO Mol Med. 2014 Feb 5;6(4):539–50. doi: 10.1002/emmm.201303214 (PMC3992079; doi:10.1002/emmm.201303214)
Supplement: Supplementary file 5 [file emmm0006-0539-sd5.pdf]

Supplementary Figure 2

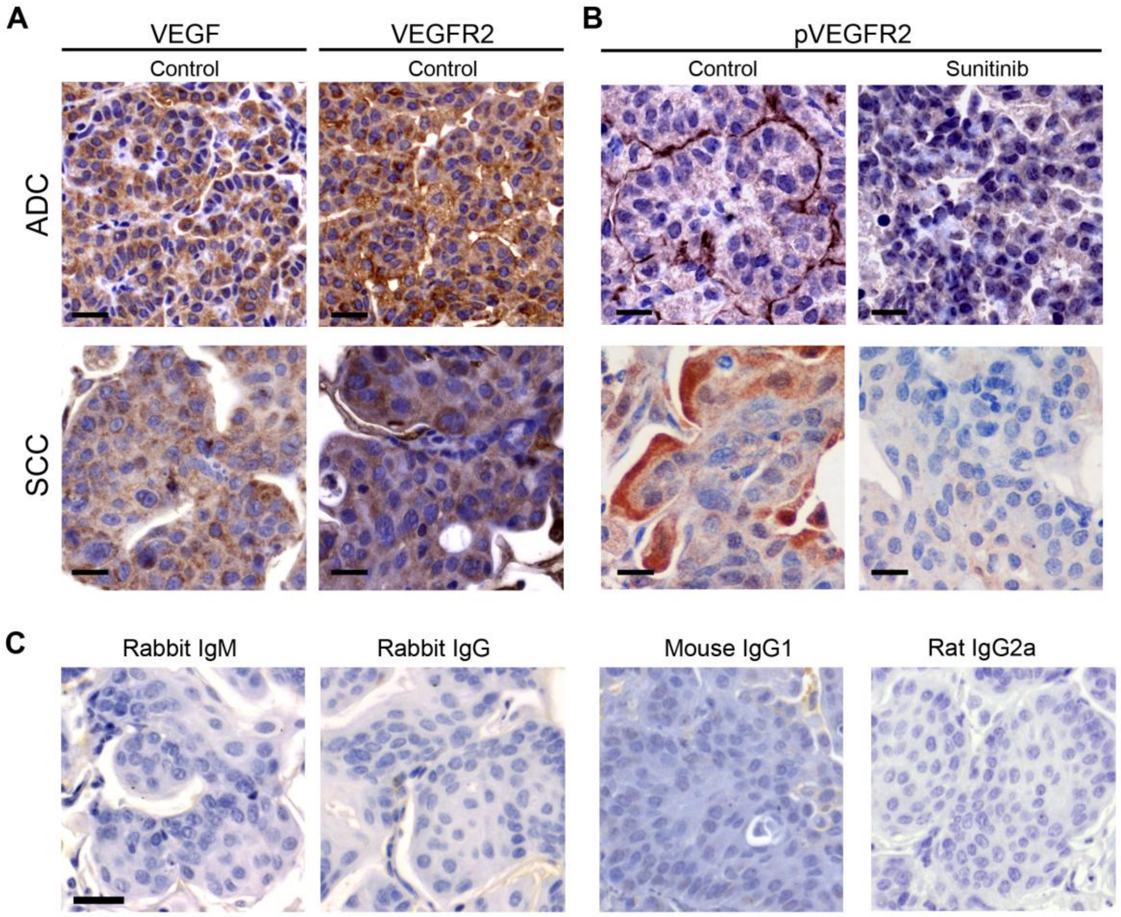

Supplementary Figure 2. VEGF, VEGFR2 and phosphoVEGFR2 (pVEGFR2) expression in mouse lung tumors. (A) Both ADC and SCC control tumors showed positive expression for VEGF and VEGFR2. (B) Immunohistochemistry for pVEGFR2 (Tyr 1175) in ADC and SCC tumors demonstrated a reduction in phosphorylation of the receptor following sunitinib treatment as compared to the control group. (B) Representative images of immunohistochemistry using isotype control antibodies. Scale bar, 25 µm.
