# Supplementary material for: Contrasting responses of non-small cell lung cancer to antiangiogenic therapies depend on histological subtype
Source: EMBO Mol Med. 2014 Feb 5;6(4):539–50. doi: 10.1002/emmm.201303214 (PMC3992079; doi:10.1002/emmm.201303214)
Supplement: Supplementary file 6 [file emmm0006-0539-sd6.pdf]

Supplementary Figure 3

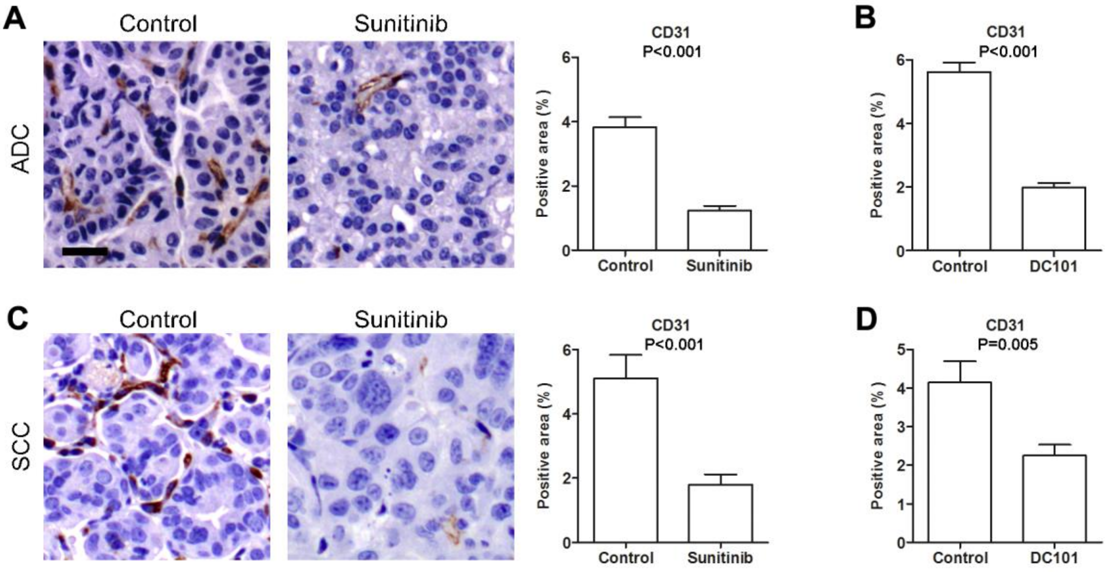

Supplementary Figure 3. Anti-VEGFR2 treatments induce vascular trimming in ADC and SCC models of lung cancer. Immunohistochemical analysis of the endothelial marker CD31 demonstrated that sunitinib (A, C) and DC101 (B, D) treatments inhibit angiogenesis in ADC (A, B) and SCC (C, D) mouse tumors. Scale bar, 25 µm. Data are presented as mean ± standard error.
