# Supplementary material for: Contrasting responses of non-small cell lung cancer to antiangiogenic therapies depend on histological subtype
Source: EMBO Mol Med. 2014 Feb 5;6(4):539–50. doi: 10.1002/emmm.201303214 (PMC3992079; doi:10.1002/emmm.201303214)
Supplement: Supplementary file 7 [file emmm0006-0539-sd7.pdf]

Supplementary Figure 4

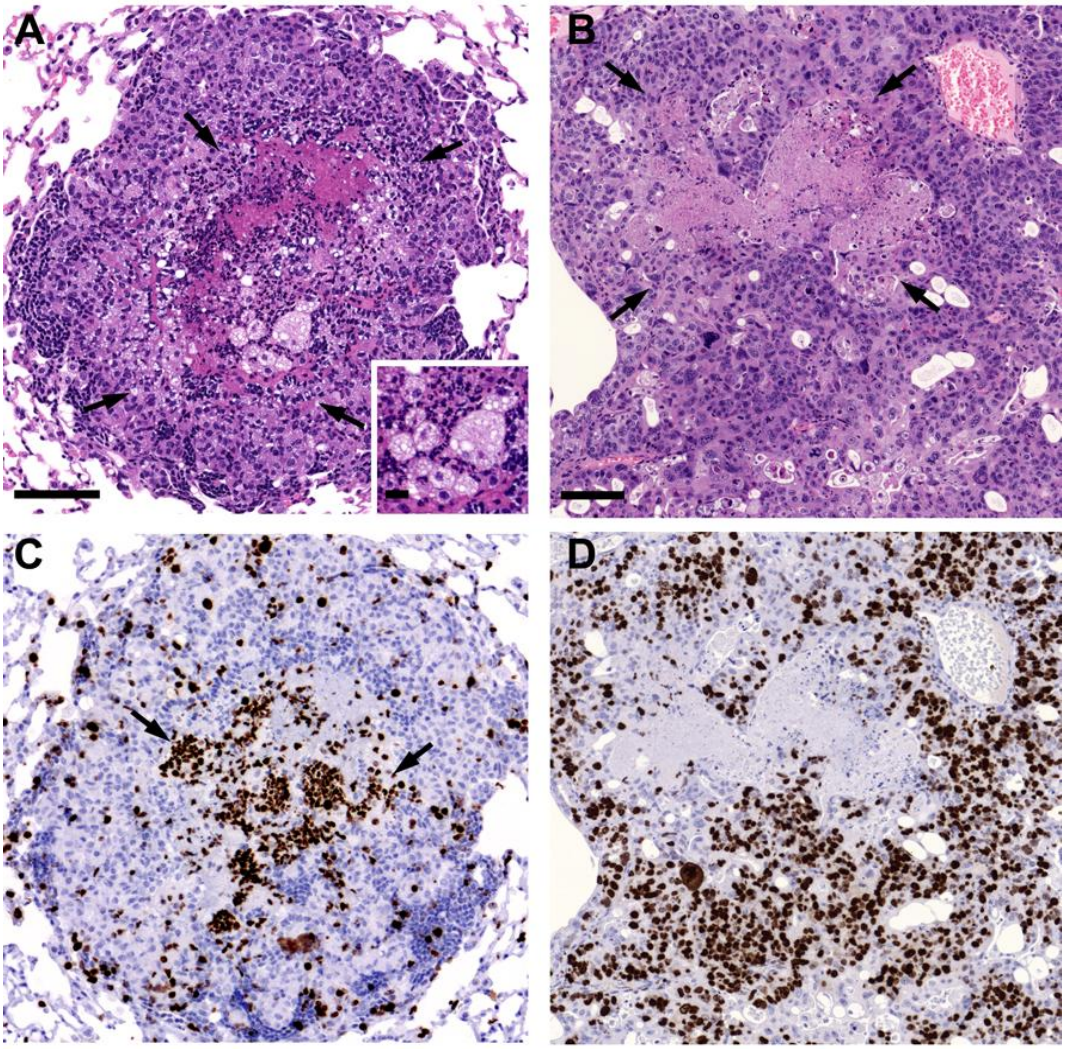

Supplementary Figure 4. Representative images of ADC and SCC tumors treated with sunitinib. (A-B) Representative H&E images of ADC (A) and SCC (B) tumors treated with sunitinib showing a central core of necrosis (arrows). Infiltrating inflammatory cells can be observed in the ADC lesion (inset). (C-D) Ki67 staining in serial sections of tumors shown in A and B. The proliferation index in sunitinib-treated ADC tumor cells is low (C) whereas inflammatory cells in the necrotic core are highly proliferative (C; arrows). It should be noted that only tumor cell expression was quantified by automatic image analysis. Conversely, Ki67 expression in sunitinib-treated SCC tumor cells treated with sunitinib (D) exhibits a high tumor proliferative rate induced by the treatment. Scale bar, 200 µm; inset, 25 µm.
