# Supplementary material for: Contrasting responses of non-small cell lung cancer to antiangiogenic therapies depend on histological subtype
Source: EMBO Mol Med. 2014 Feb 5;6(4):539–50. doi: 10.1002/emmm.201303214 (PMC3992079; doi:10.1002/emmm.201303214)
Supplement: Supplementary file 8 [file emmm0006-0539-sd8.pdf]

Supplementary Figure 5

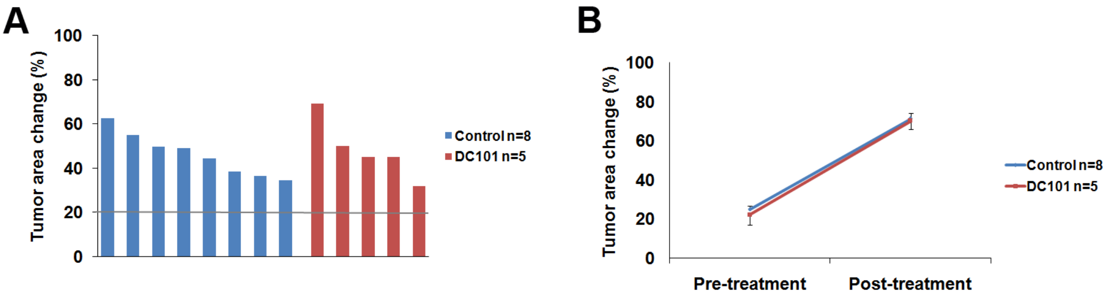

Supplementary Figure 5. DC101 therapy induced progression of the disease in early NTCU-induced SCC bearing mice. Mice receiving DC101 therapy at early stage of the disease (8 weeks of NTCU treatment) showing progression of the disease. (A) Waterfall plot and (B) line plot of tumor response to DC101 treatment at early stage of the disease in the NTCU-induced SCC mouse model. Data represent the mean tumor area ± standard error.
