# Supplementary material for: Contrasting responses of non-small cell lung cancer to antiangiogenic therapies depend on histological subtype
Source: EMBO Mol Med. 2014 Feb 5;6(4):539–50. doi: 10.1002/emmm.201303214 (PMC3992079; doi:10.1002/emmm.201303214)
Supplement: Supplementary file 9 [file emmm0006-0539-sd9.pdf]

Supplementary Figure 6

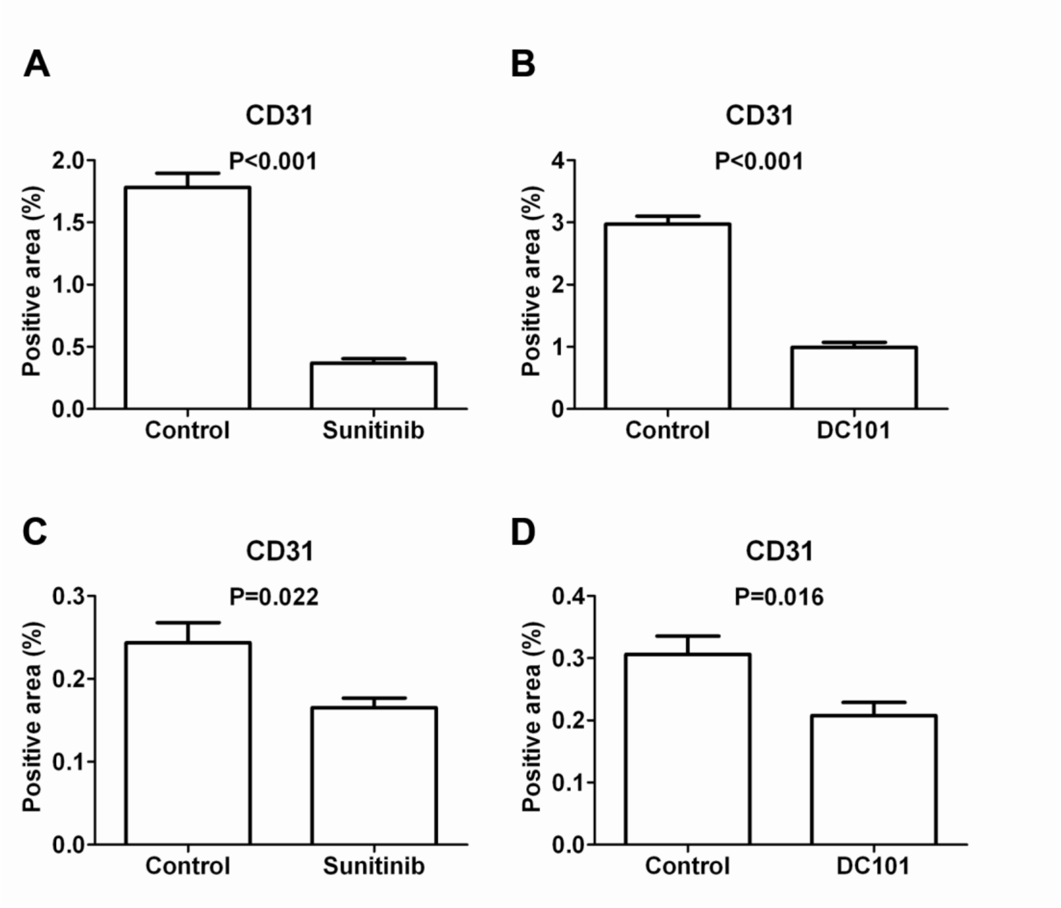

Supplementary Figure 6. Antiangiogenic treatments induce vascular trimming in ADC and SCC tumorgraft models. Immunohistochemical analysis of the endothelial marker CD31 demonstrated that sunitinib- (A, C) and DC101- (B, D) treatments inhibit angiogenesis in ADC (A, B) and SCC (C, D) mouse tumors. Data are presented as mean ± standard error.
