# Supplementary material for: Contrasting responses of non-small cell lung cancer to antiangiogenic therapies depend on histological subtype
Source: EMBO Mol Med. 2014 Feb 5;6(4):539–50. doi: 10.1002/emmm.201303214 (PMC3992079; doi:10.1002/emmm.201303214)
Supplement: Supplementary file 10 [file emmm0006-0539-sd10.pdf]

Supplementary Figure 7

A

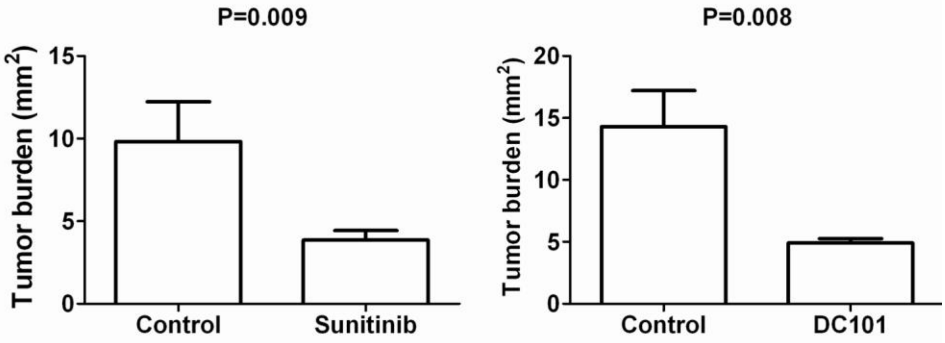

B

	Liver micrometastasis		Spleen micrometastasis		Kidney micrometastasis	
	**No**	**Yes**	**No**	**Yes**	**No**	**Yes**
**Control**	1	5	0	6	1	5
**Sunitinib**	0	6	2	4	3	3
**Control**	1	5	1	5	3	3
**DC101**	4	2	3	3	3	3

Supplementary Figure 7. Anti-VEGFR2 treatments reduce tumor invasion of the lungs. (A) The metastatic tumor area in the lungs of UN-ADC12 tumorgraft bearing mice was reduced after sunitinib or DC101 treatments. Data are presented as mean ± standard error; n=6 for all groups. (B) Contingency table listing the number of animals in which micrometastases were found.
