# Supplementary material for: Contrasting responses of non-small cell lung cancer to antiangiogenic therapies depend on histological subtype
Source: EMBO Mol Med. 2014 Feb 5;6(4):539–50. doi: 10.1002/emmm.201303214 (PMC3992079; doi:10.1002/emmm.201303214)
Supplement: Supplementary file 11 [file emmm0006-0539-sd11.pdf]

Supplementary Figure 8

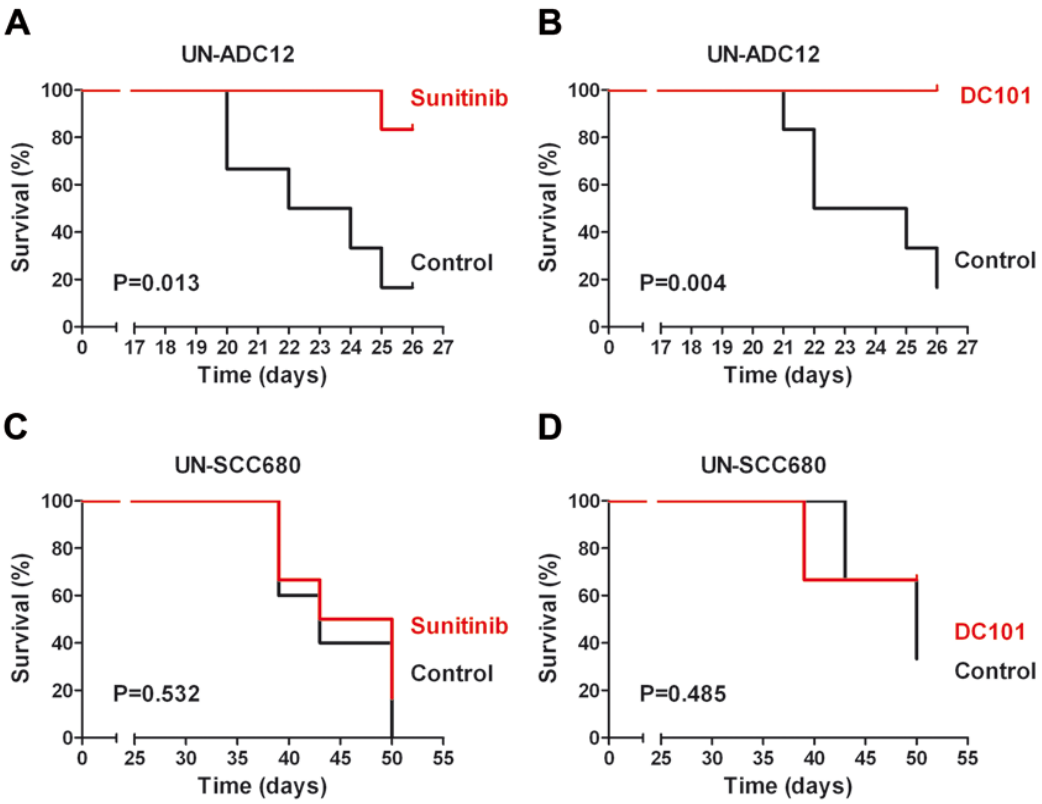

Supplementary Figure 8. Contrasting effects of VEGFR2 blockade on overall survival according to tumor histology. Kaplan-Meier survival curves of immunodeficient mice bearing UN-ADC12 tumorgrafts (A-B) and UN-SCC680 tumorgrafts (C-D) treated with sunitinib (A, C) or DC101 (B, D). The proportion of mice reaching experimental endpoint (accomplished when tumors reached 1.7 cm in diameter) over time is shown. P-values are those associated with the log-rank test; n=6 for all groups.
