# Supplementary material for: Contrasting responses of non-small cell lung cancer to antiangiogenic therapies depend on histological subtype
Source: EMBO Mol Med. 2014 Feb 5;6(4):539–50. doi: 10.1002/emmm.201303214 (PMC3992079; doi:10.1002/emmm.201303214)
Supplement: Supplementary file 12 [file emmm0006-0539-sd12.pdf]

Supplementary Figure 9

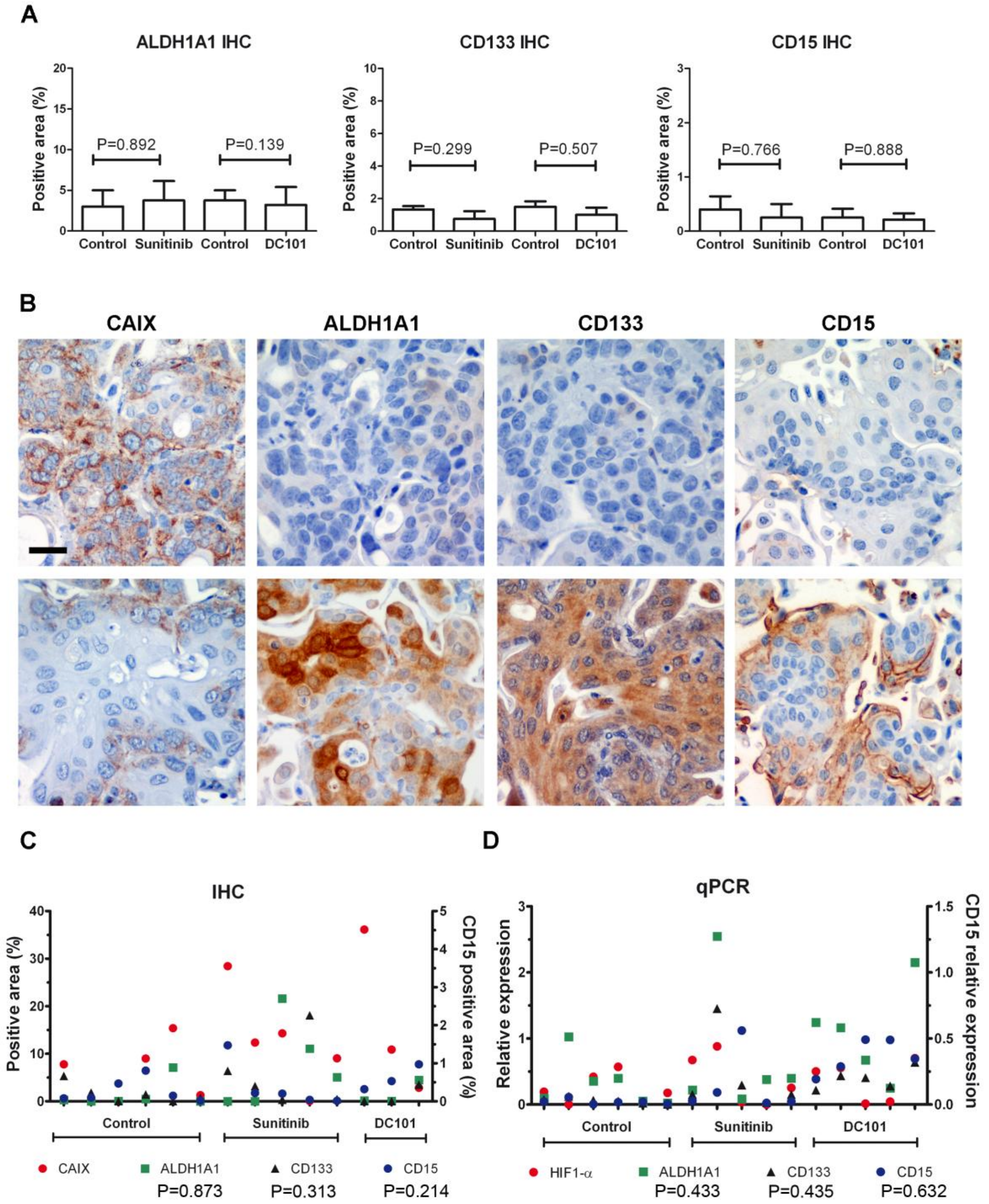

Supplementary Figure 9. The expression of hypoxic markers does not correlate with the expression of stem cell markers. (A) Immunohistochemical (IHC) relative expression of ALDH1A1, CD133, and CD15 stem cell markers in urethane-induced ADC tumors. Data are presented as mean ± standard error. (B) Representative immunohistochemistry images of two SCC control tumors showing different expression of CA-IX and stem cell markers ALDH1A1, CD133, and CD15 in serial sections. Scale bar, 25 µm. (C) Automatic quantification of CA-IX and stem cell markers ALDH1A1, CD133, and CD15 immunohistochemical (IHC) expression in serial sections. (D) The relative expression of HIF-1α, ALDH1A1, CD133, and CD15 was analyzed by real time quantitative PCR (qPCR) in SCC-tumor bearing mice. (C-D) CD15 expression is represented in the right axis. P-values for the correlation (Spearman rank test) between CAIX or HIF1-α and stem cell markers are shown.
