# Supplementary material for: Contrasting responses of non-small cell lung cancer to antiangiogenic therapies depend on histological subtype
Source: EMBO Mol Med. 2014 Feb 5;6(4):539–50. doi: 10.1002/emmm.201303214 (PMC3992079; doi:10.1002/emmm.201303214)
Supplement: Supplementary file 13 [file emmm0006-0539-sd13.pdf]

Supplementary Figure 10

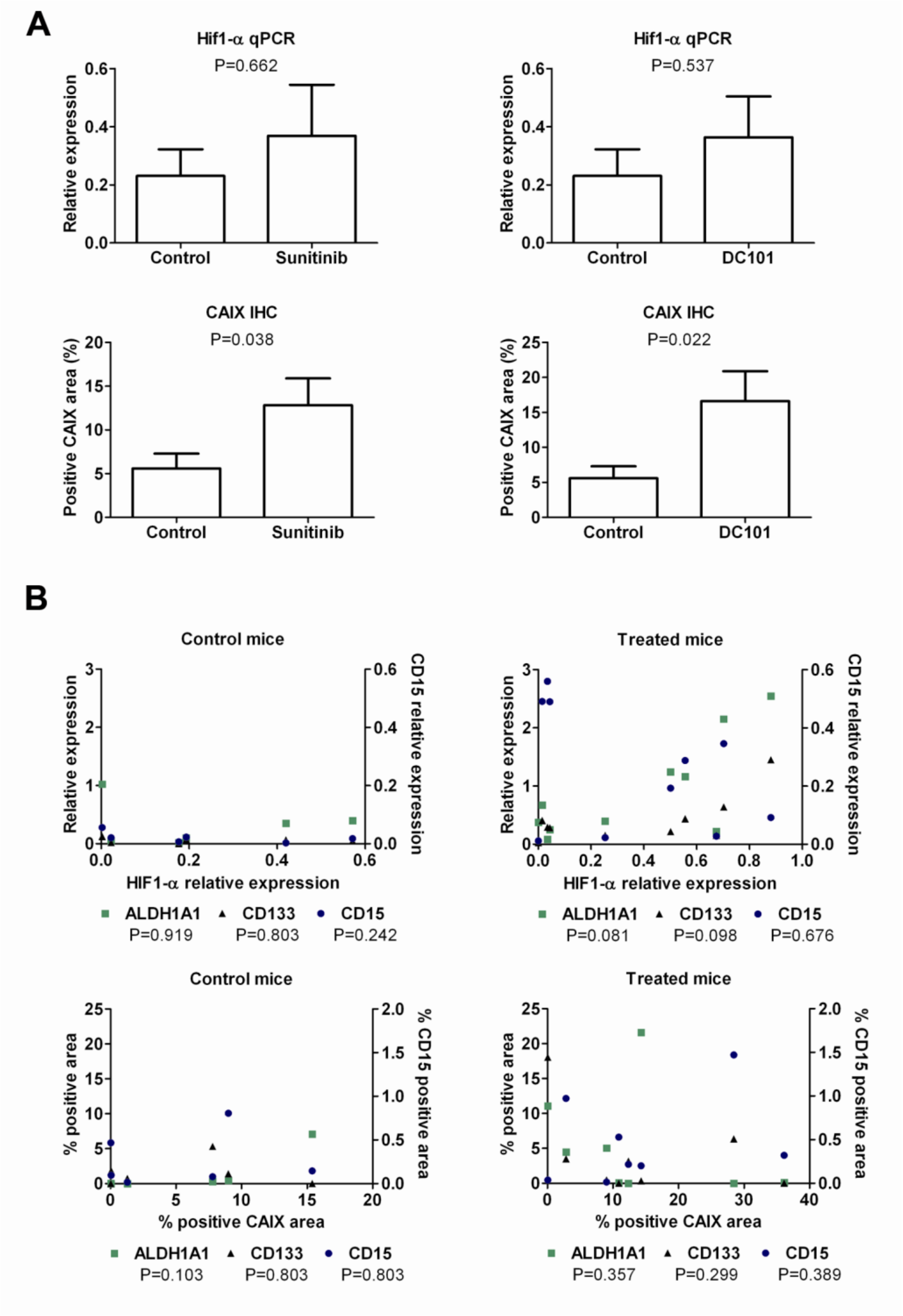

Supplementary Figure 10. Differential expression of hypoxic markers and stem cell markers between groups. (A) HIF-1α and CAIX expression was compared between control and treated mice. (B) Correlation between HIF-1α or CAIX and the expression of stem cell markers ALDH1A1, CD133, and CD15. P values for the correlation (Spearman rank test) between CAIX or HIF1-α and stem cell markers are shown.
