# Supplementary material for: Contrasting responses of non-small cell lung cancer to antiangiogenic therapies depend on histological subtype
Source: EMBO Mol Med. 2014 Feb 5;6(4):539–50. doi: 10.1002/emmm.201303214 (PMC3992079; doi:10.1002/emmm.201303214)
Supplement: Supplementary file 14 [file emmm0006-0539-sd14.pdf]

Supplementary Table 1: Primer sequences.

Primers were obtained from Sigma.

Gene	Forward (5´to 3´)	Reverse (5´to 3´)
**ALDH1A1**	GCACTCAATGGTGGGAAAGT	GGCCACACACTCCAATAGGT
**CD133**	GCAAAGAGCAATTTCAGAGACTTTC	CCTTGTTCTTGGTGTTGGTGTACT
**CD15**	TGGTGGCCTGGGTTGTG	CGACTCAGCTGGTGGTAGTAACG
**HIF-1α**	GTTTACTAAAGGACAAGTCAC	TTCTGTTTGTTGAAGGGAG
**GUSB**	CTCTGGTGGCCTTACCTGAT	CTCAGTTGTTGTCACCTTCACC
