# Supplementary material for: Contrasting responses of non-small cell lung cancer to antiangiogenic therapies depend on histological subtype
Source: EMBO Mol Med. 2014 Feb 5;6(4):539–50. doi: 10.1002/emmm.201303214 (PMC3992079; doi:10.1002/emmm.201303214)
Supplement: Supplementary file 15 [file emmm0006-0539-sd15.pdf]

Supplementary material

Contrasting Responses of Non-Small Cell Lung Cancer to Antiangiogenic Therapies depend on histological subtype

Marta Larrayoz[1,2], Ruben Pio[1,3], María J. Pajares[1,2], Isabel Zudaire[1,3], Daniel Ajona[1], Oriol Casanovas[4], Luis M. Montuenga[1,2] and Jackeline Agorreta[1,2]

Table of contents:

Supplementary Table 1: Primer sequences. 2

SUPPLEMENTARY FIGURES 3

Supplementary Figure 1. Histological evaluation of chemically-induced murine lung tumors. 3

Supplementary Figure 2. VEGF, VEGFR2 and phosphoVEGFR2 (pVEGFR2) expression in mouse lung tumors 4

Supplementary Figure 3. Anti-VEGFR2 treatments induce vascular trimming in ADC and SCC models of lung cancer 5

Supplementary Figure 4. Representative images of ADC and SCC tumors treated with sunitinib. 6

Supplementary Figure 5. DC101 therapy induced progression of the disease in early NTCU-induced SCC bearing mice. 7

Supplementary Figure 6. Antiangiogenic treatments induce vascular trimming in ADC and SCC tumorgraft models. 8

Supplementary Figure 7. Anti-VEGFR2 treatments reduce tumor invasion of the lungs. 9

Supplementary Figure 8. Contrasting effects of VEGFR2 blockade on overall survival according to tumor histology. 10

Supplementary Figure 9. The expression of hypoxic markers does not correlate with the expression of stem cell markers. 11

Supplementary Figure 10. Differential expression of hypoxic markers and stem cell markers between groups. 12
